# A Bibliometric Analysis and Visualization of Decision Support Systems for Healthcare Referral Strategies

**DOI:** 10.3390/ijerph192416952

**Published:** 2022-12-16

**Authors:** Hesham Ali Behary Aboelkhir, Adel Elomri, Tarek Y. ElMekkawy, Laoucine Kerbache, Mohamed S. Elakkad, Abdulla Al-Ansari, Omar M. Aboumarzouk, Abdelfatteh El Omri

**Affiliations:** 1 College of Science and Engineering, Hamad Bin Khalifa University, Doha 34110, Qatar; 2 Department of Mechanical and Industrial Engineering, College of Engineering, Qatar University, Doha 2713, Qatar; 3 Surgical Research Section, Department of Surgery, Hamad Medical Corporation, Doha 3050, Qatar; 4 College of Medicine, QU-Health, Qatar University, Doha 2713, Qatar; 5 School of Medicine, Dentistry and Nursing, The University of Glasgow, Glasgow G12 8QQ, UK

**Keywords:** healthcare referral, medical transfer, quality of care, patient pathways, decision support systems, operations management

## Abstract

Background: The referral process is an important research focus because of the potential consequences of delays, especially for patients with serious medical conditions that need immediate care, such as those with metastatic cancer. Thus, a systematic literature review of recent and influential manuscripts is critical to understanding the current methods and future directions in order to improve the referral process. Methods: A hybrid bibliometric-structured review was conducted using both quantitative and qualitative methodologies. Searches were conducted of three databases, Web of Science, Scopus, and PubMed, in addition to the references from the eligible papers. The papers were considered to be eligible if they were relevant English articles or reviews that were published from January 2010 to June 2021. The searches were conducted using three groups of keywords, and bibliometric analysis was performed, followed by content analysis. Results: A total of 163 papers that were published in impactful journals between January 2010 and June 2021 were selected. These papers were then reviewed, analyzed, and categorized as follows: descriptive analysis (*n* = 77), cause and effect (*n* = 12), interventions (*n* = 50), and quality management (*n* = 24). Six future research directions were identified. Conclusions: Minimal attention was given to the study of the primary referral of blood cancer cases versus those with solid cancer types, which is a gap that future studies should address. More research is needed in order to optimize the referral process, specifically for suspected hematological cancer patients.

## 1. Background

**Figure 1:**
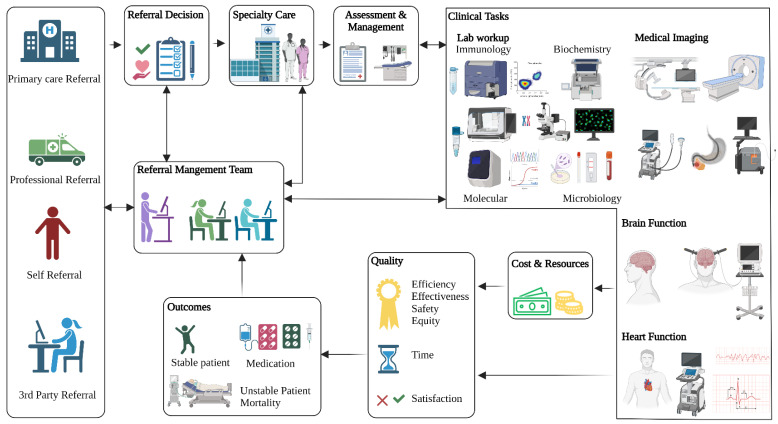
Example of referral pathways in healthcare. Created with BioRender.com.

The referral process is critical to healthcare and begins when a primary care physician (PCP) believes that the patient may require a specialist examination. The PCP refers the patient to a specialist with a referral letter that provides a detailed summary of the patient’s medical history and any additional information that is required for a smooth transition of care. While a third of patients are referred to specialists each year in the United States [1], the process is often complex and inefficient [2]. The referral pathway resembles a health service supply chain, causing delays to patient care that cost both lives and money (Figure 1). The process requires good communication and collaboration between all three parties: the PCPs, the specialists, and the patients [3]. Data and information transfer between parties is necessary in order to find common ground and to achieve the following primary goals of the referral process: accuracy, timeliness, effectiveness, and equal access to care [4,5]. Information delivery can occur through a traditional referral letter [6] or via an electronic record [7]. Operation management ensures that the patients can reach the specialist and receive the proper care at the right time. Indeed, access to a specialist is a vital performance measure for the quality of comprehensive health care management [8]. Managing access through scheduling and triaging patients is a central task in this process [9].

This literature review focuses on the primary referral for a suspected cancer patient from a PCP to a specialist, as this is often the trigger step in the healthcare pathway. Several manuscripts have shown that much of the reduction in cancer mortality is attributed to earlier diagnosis [10,11]. The cancer burden continues to grow worldwide, exerting emotional, physical, and financial strain on the individuals, the families, the communities, and the health systems (World Health Organization, 2020) [12]. Despite efforts to improve the frequency and the quality of referrals, this is a complex field that requires additional analysis.

Over the past decade, there has been a growing interest in solving problems using innovative operations management (OM) techniques and technologies, including simulation and optimization models. These models identify the possible causes of a problem, recommend appropriate interventions, and predict the consequences of certain decisions. This has made operation management approaches a base for many future research directions (Section 5.2).

To the best of our knowledge, the novelty of this manuscript is its use of a hybrid structured bibliometric analysis approach in order to provide a comprehensive understanding of the entire body of research on the referral process in health care (RPHC). The last relevant papers that reviewed the problems and solutions that are associated with the primary referral process covered articles that were written before 2015 [13,14]. While Greenwood-Lee’s review was published in 2018, it only included articles that were published before 2015, which left out the studies that covered new techniques, such as the simulation and optimization models of the referral process. The current review aims to fill the gaps with updated articles covering a wider range of topics (Section 3.2). The importance of the hybrid model is that it makes the combination of a quantitative bibliometric review with a qualitative structured review possible. This allows for the quantitative evaluation of the scientific literature, in addition to an understanding of the similarities and differences among them [15]. This review uses the latest bibliometric analysis methods with a structured review in order to comprehensively study the RPHC from multiple aspects and combined paradigms.

## 2. Methodology

A mixed review of bibliometric and content analysis was used to capture the knowledge structure of past works, present trends, and anticipated future research. The studies covered a wide range of goals, objectives, methodologies, techniques, and models to assess RPHC. A broad research scope was used in order to cover these different perspectives, while specifically focusing on cancer patient referrals.

**Figure 2:**
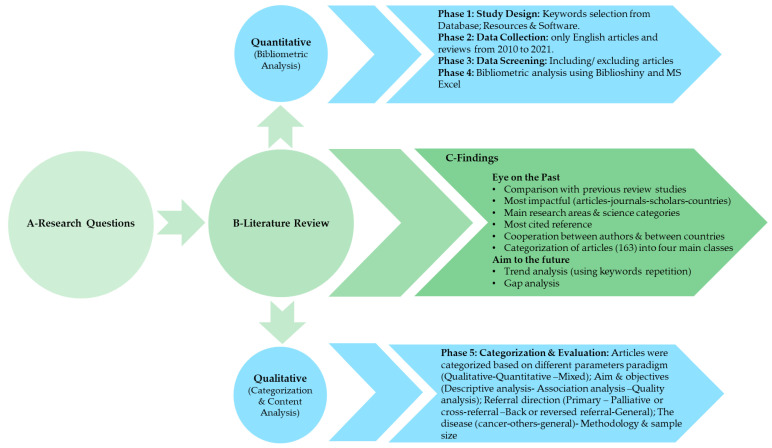
The methodology flow chart.

Since bibliometric analysis is primarily quantitative, qualitative content analysis was also conducted. Manuscripts were extracted from the following electronic databases: Web of Science, Scopus, and PubMed. The analysis had the following five phases, as shown in Figure 2: (1) study design, (2) data collection, (3) initial data analysis, (4) Bibliometric analysis, and (5) qualitative analysis.

### 2.1. Phase 1: Study Design

In the first phase, keyword groups and filtering operators were identified. The search strategy was kept broad and used terms that included the primary keywords in order to avoid missing relevant papers. The exact keywords were searched in Web of Science, Scopus, and PubMed, as illustrated in Table 1. The keywords consisted of three groups of synonymous words. These groups were connected with “AND” as a filtering operator. The searches for these words were explored within the article title, the abstract, and the keywords.

### 2.2. Phase 2: Data Collection

The first step in the second phase was to determine the time span, beginning with an unrestricted time frame. The number of articles was found to increase annually, beginning in 2010. Thus, articles from January 2010 to June 2021 were included in order to capture the newest articles relating to RPHC. After many trials, the keywords that captured the most relevant articles were chosen, using Boolean operators “AND” and “OR” to concentrate the research. Keywords, such as tumors, cancer, blood disease, lymphoma, leukemia, and multiple myeloma, were added to collect the most significant number of articles specific to cancer care referrals. In addition, initial searches yielded more articles about solid cancers, such as colorectal, breast, and lung cancers, than articles about blood cancers.

### 2.3. Phase 3: Initial Data Analysis

**Figure 3:**
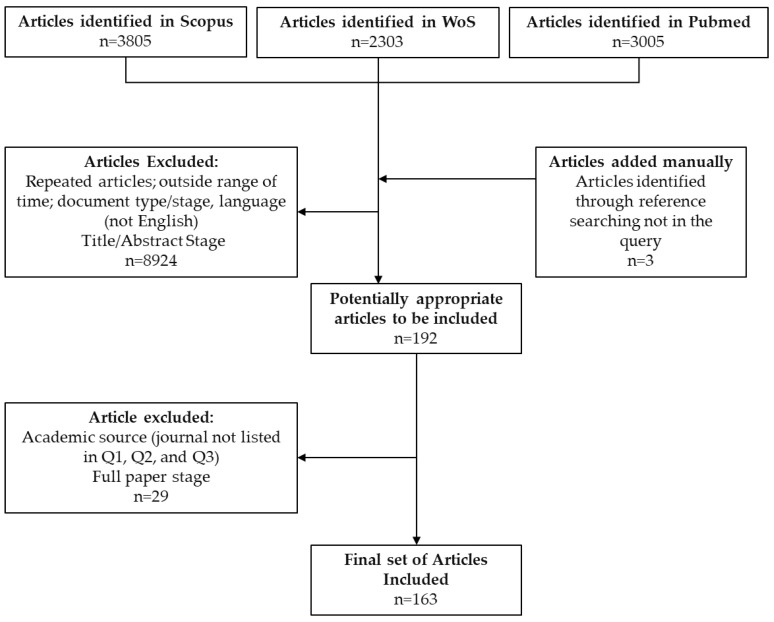
Flowchart of items included in the review.

The research results of the three databases were collected and filtered using Microsoft Excel in order to exclude repetition. The final list of articles was revised by excluding the articles that were not deemed relevant based on their titles and abstracts (Figure 3). Papers from journal sources that were ranked below the third quartile using the most recent IF Clarivate Analytics JCRI 2021 were also excluded (Table S1). Three articles were reviewed from the reference lists of the relevant papers and were included in the study.

### 2.4. Phase 4: Bibliometric Analysis (Quantitative)

The primary task was to select the bibliographic database and analytic software tool. Web of Science was chosen as the core bibliographic data source and was found to cover most of the relevant articles (158/163). The second step involved a review of the available software tools for bibliometric analysis. The Biblioshiny Library was selected for its reliability in the processing and collection of bibliographic data [16].

Bibliometric analysis was performed using Biblioshiny software, which was developed by Massimo and Cuccurullo (2017). It is a user-friendly tool that can generate different comparisons and visualizations with high validity, including historic analysis, keywords analysis, collaboration mapping, and thematic analysis. Cooperation between authors were assessed using Excel.

### 2.5. Phase 5: Categorization and Evaluation (Qualitative)

The articles were then categorized using qualitative, quantitative, or mixed paradigms. Depending on the aims and objectives of the study being assessed, descriptive, association (root cause), interventions, or quality analysis was performed. These categories were divided into additional subcategories, such as the interventions category, which was divided into modeling and non-modeling. The articles were further differentiated based on the type or the direction of the referral, as follows: primary, reverse, cross, palliative, or general. The type of disease and methodology were also used as parameters for the categorization.

## 3. Results and Discussion

This section begins with results from the quantitative analysis, including an analysis of the history of RPHC, followed by an overview of the referral trends. The results from the qualitative analysis are then discussed after classifying and evaluating the articles by topic.

### 3.1. Descriptive Bibliometric Analysis of RPHC Publications

The database searches revealed 163 articles that covered the topic of RPHC from January 2010 to June 2021. Out of these, 158 articles were found in Web of Science, and 5 were found in Scopus. All of these articles were included in the quantitative analysis in order to assess the patterns in RPHC over the past decade. Google scholar was used to unify the citation numbers between the articles that were collected from the different databases. The results showed an increasing pattern of producing new articles and using these as references for other articles, as shown by the citation number (Table 2).

**Figure 4:**
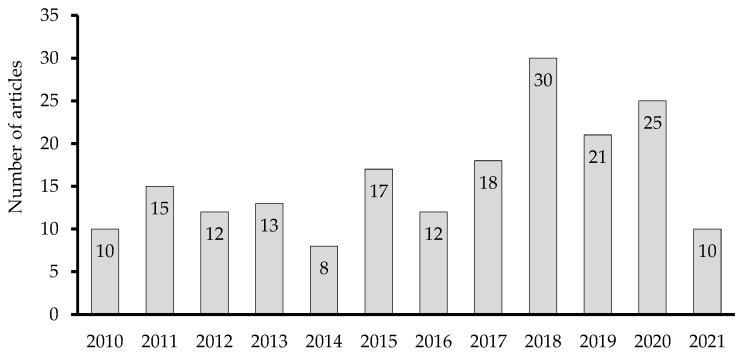
Annual publication distribution, 2010–2021 (n = 163 papers).

The growing number of articles that are published on RPHC each year, the established knowledge-base on this topic, and the new trends and opportunities, make this an exciting field in which to work (Figure 4).

#### 3.1.1. Comparison with Prior Reviews of RPHC

While 10% of the papers that are included in this analysis were systematic reviews (n = 16), most of them were concentrated on only one of the performance measures, such as improving the waiting time or receiving an early diagnosis [17,18,19,20]. Other manuscripts highlighted the issue of cooperation between the service providers [21], focused on one intervention, such as the use of an electronic consultation service [22] or letters to improve the referral process [23], or highlighted some specific type of referral, such as cross and palliative referral [24,25,26].

Additionally, some of the studies reviewed the impact of risk factors, such as low income, age, and medical situation, on referral outcomes [27,28]. One systematic review assessed the performance measures of the referral process in order to identify those that are associated with specialty referrals [29]. Blank L et al. (2014) and Greenwood-Lee et al. (2018) reviewed the issues and the solutions of the primary referral process, which was the main scope of the current review [13,14]. While Greenwood-Lee’s review was published in 2018, it only included the articles that were published before 2015, which left out the studies that covered new techniques, such as simulation and optimization models of the referral process. The current review aims to fill the gaps with updated articles covering a wider range of topics (Table 3). This study is innovative because it combines the operation management approach with the healthcare service approach in the referral process (Section 4).

#### 3.1.2. Most Impactful Articles

This study used the citation numbers to determine the degree of an article’s impact. The citation number was checked per year in order to disregard the time variation factor, and the Google Scholar citation number was used to unify this index across the two databases. Table 4 shows the most impactful papers and their rank, relying on the citation number and the citation per year ratio.

Out of the top ten articles, four were reviews indicating that the current paper is likely to contribute to the RPHC knowledge base and provide a good reference for future studies. Five out of the ten most-cited articles studied the referral process in cancer care, which is likely because of the seriousness of this disease and the consequences of referral delays. The work by Lyratzopoulos G. et al. studied the number of PCP consultations before referral and the factors of its variations. It was the most-cited article, with 348 citations [32]. The paper by Sud A. et al., which examined the effect of the COVID-19 pandemic in the cancer referral pathway and cancer survival [11], had an overall rank of five, but had the highest number of citations per year, which is likely because the COVID-19 pandemic has generated a large field for research. One of the primary issues of the pandemic is its impact on healthcare referral. Overall, the most prominent and influential authors, the most contributing journal sources, and the most productive countries were identified by this bibliometric analysis.

#### 3.1.3. Main Research Contributors

Using the Hirsch index (H-Index) and the average number of citations per document, a top 10 list of authors who contributed to RPHC was identified from our dataset (Table 5).

The H-index is an author-level metric measuring publication productivity and the citation impact of scholars [38].

Lyratzopoulos G. was ranked first with, an H-index of five. This author published seven articles in RPHC from 2010 to 2021, and each of his manuscripts was cited 31 times on average. His article, which ranked first in the top-cited articles, was co-published by three of the top ten authors: Lyratzopoulos G., Rubin G., and Abel G.A.

Abel G.A. was ranked second, with an H-index of five. He contributed to six articles, and each manuscript was cited 29.7 times on average. His articles focused on the referral process in cancer, particularly blood cancer care, using a survey as the primary data collection tool [39].

Vargas I. was ranked third with an H-index of three. This author contributed to five articles, and each manuscript was cited 46.8 times on average. This article reviewed clinicians’ opinions on the contribution of coordination mechanisms to improving clinical coordination between primary and secondary care in Catalonia [40]. The cooperation between the authors will be assessed in Section 3.3.2.

##### Top 20 Journals including RPHC Articles

**Figure 5:**
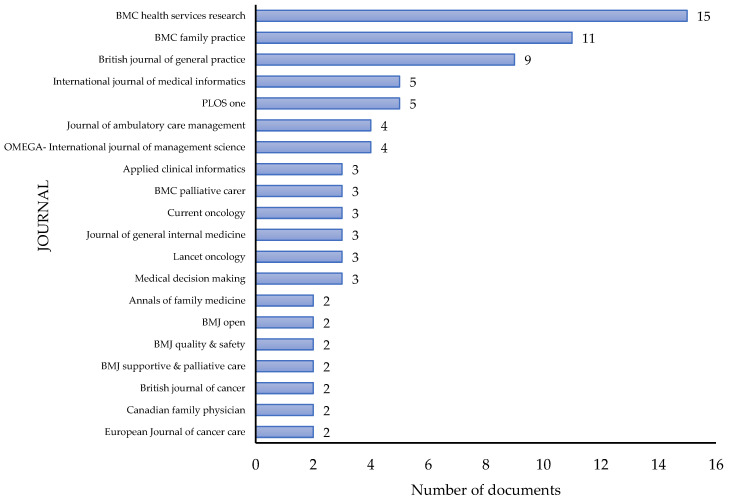
Top journal contributions (most relevant) to RPHC publications.

BMC Health Service Research is ranked first among the most relevant sources because it contained the highest number of articles in our study (15). BMC Family Practice was ranked second, with eleven articles, and British Journal of General Practice was ranked third, with nine articles. Using the total number of citations as the parameter for ranking the contributing journals, The Lancet Oncology Journal was ranked first by a large margin, with 391 total citations. However, this journal only contributed to three articles in this study, which is likely due to its clinical focus (Figure 5).

##### Country-Specific Contributions

**Figure 6:**
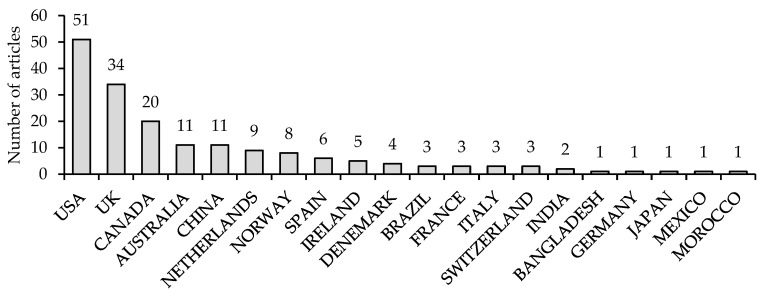
Country of origin of authors with the highest article contribution.

The countries producing the highest number of research articles on RPHC were the United States (51 articles), the United Kingdom (34 articles), and Canada (20 articles) (Figure 6).

The negligible contribution of the Middle East makes it important to fill the knowledge gap through a review paper that acts as a base for subsequent studies of the referral process and the healthcare system in that region (Figure 6)

#### 3.1.4. Main Research Areas and Science Categories

This study shows the leading research areas, as well as any potential research gaps. Web of Science categorizes articles using science categories. Table 6 shows the diverse research areas that have been covered by this review, including the number and the percentage of articles in each category.

Only six articles (4%) focused on operational research, and five of them were published in the last two years, indicating a new trend of modeling solutions using optimization and simulation to handle the problems with the referral process [41,42]. This indicates a research gap and opportunities to use these techniques in future research. This will be discussed in more detail in Section 5.

### 3.2. Historic Analysis of RPHC Papers

#### Highest Cited References

**Figure 7:**
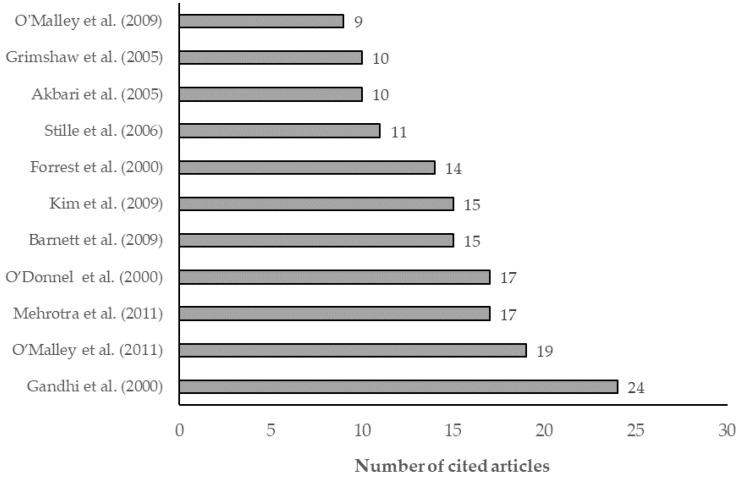
Most-cited references [1,3,33,43,44,45,46,47,48,49,50].

Biblioshiny was used to identify the references with the most highly cited articles (Figure 7) in order to identify the scientific base from which research has been launched within the last decade.

(Gandhi T.K. et al., 2000) was cited in 24 articles and was used as an essential reference for research in primary and specialty care subfields that focused on communication breakdown during the outpatient referral process [43].

(O’Malley A.S., 2011) was cited in 19 articles, along with the 2009 manuscript by O’Malley et al. This manuscript assessed the communication quality between the primary care and the specialist physicians [3].

(Mehrotra A, 2011) was cited by 17 articles. This descriptive study analyzed the referral patterns in the United States [33].

(O’Donnell CA, 2000) was cited by 17 articles. This article audited the referral rates in comparison to the National Institute for Clinical Excellence (NICE) referral guidelines [44].

(Barnett M.L., 2012) was cited by 15 articles, and it covered the national trends in physician referrals from 1999 to 2009; it is a significant source of secondary data [45].

(Kim Y. 2009) was cited by 15 articles, and his study analyzed whether electronic referrals can improve subspecialty care access in safety-net settings [46].

(Forrest C.B., 2000) was cited by 14 articles, and he reported how physicians coordinate patient care for specialty referrals and examined the impact on the satisfaction of referring physicians [51].

### 3.3. Keywords and Trend Analysis

#### 3.3.1. Keyword Analysis

A keyword analysis was performed in order to define trends in particular RPHC topics. Keywords aid in the discovery of specific information during literature searches, helping to connect the topics that are being searched with the research content. Cluster analysis was performed in order to identify the most repeated five keywords (term) per year that featured the trend of topics per year.

**Figure 8:**
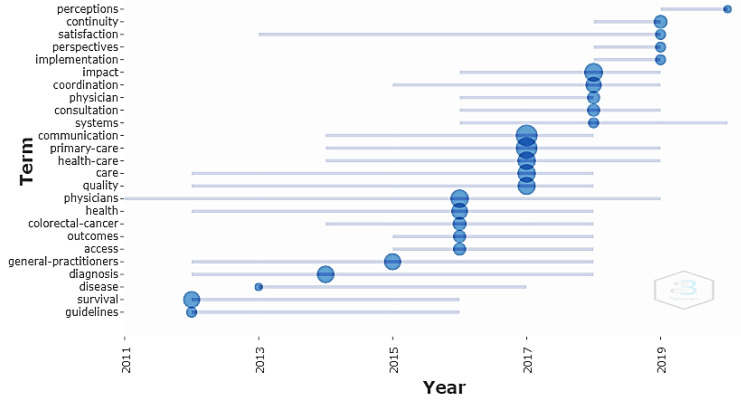
Trend topics using word repetition.

Figure 8 shows the most repeated keywords in the article titles each year. This analysis shows how particular keywords have evolved. The blue circles show the most repeated keywords in a particular year, and the circle area indicates the number of articles that were discovered using that keyword.

Articles that were published before 2016 frequently focused on general practitioner adherence to guidelines that concentrated on early disease diagnosis and the impact on survival. During 2016, the trend evolved to include sharing physicians, managing access to specialists, and the outcomes of delayed referral, especially for colorectal cancer. In 2017, the publications focused on the communication between the health care providers and the total quality of the referral process. New communication methods were tested, including the latest communications technology, mobile applications, electronic referrals, and electronic medical reports. From 2018 onwards, the studies transitioned from being process-oriented to patient-oriented, as can be seen from the high usage of terms such as perception, satisfaction, and perspective. Additionally, the selection was extended to include the patient in the referral decision-making process and to apply the findings to new referral models. By 2020, most of the publications focused on coordination across the health facilities and cooperation between caregivers, resulting from the COVID-19 pandemic impact on the healthcare system.

#### 3.3.2. Cooperation between Authors and Countries

**Figure 9:**
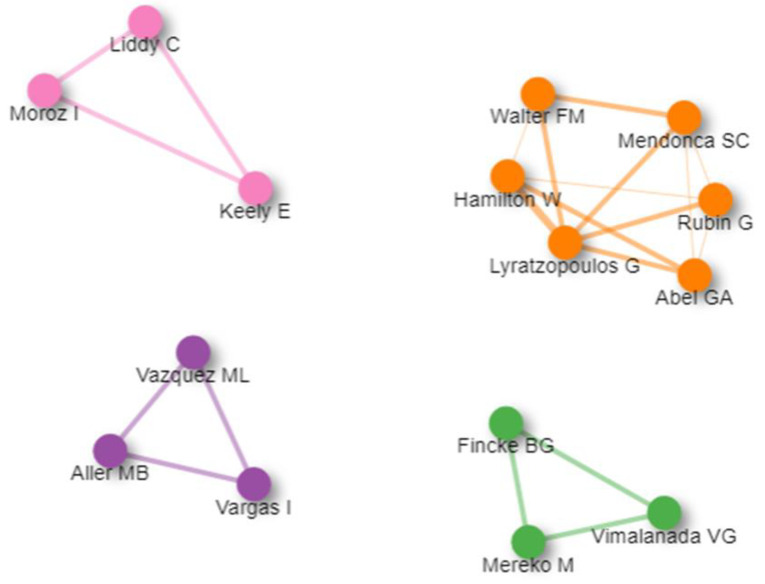
Authors’ collaboration network.

This review studied the cooperation between authors in joint research and its impact on their research. There are four main clusters of collaboration networks. The orange cluster leads them with six authors (Lyratzopoulos G., Abel G.A., Hamilton W., Walter F.M., Mendonca S.C., and Rubin G.), who collaborated in their work. This cluster collaboration generated the most-cited publication in that field. In the pink cluster, three authors (Moroz I., Liddy C., and Keely E.) collaborated in order to publish three papers in that field. Liddy C. was the first author in all of them. Vargas I. worked with Vazquez ML. in the purple cluster in four of the articles. In addition, he and Vazquez M.L. were the co-authors of Aller M.B.’s two articles. In the green cluster, Vimalanda VG., Fincke BG., and Mereko M. collaborated in two papers. Moreover, Vimalanda V.G. collaborated with each one of the other authors in an extra article (Figure 9).

**Figure 10:**
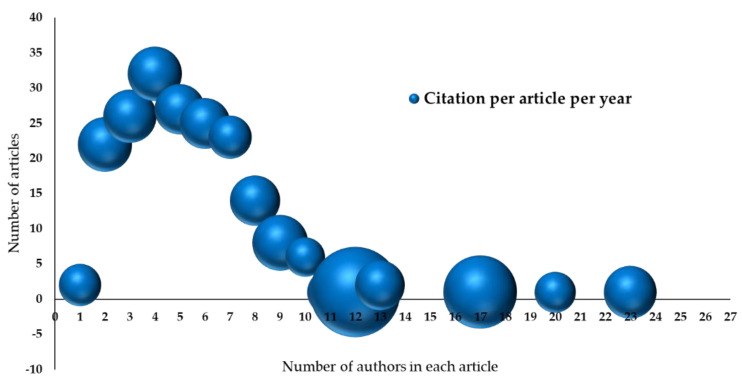
Cooperation between authors.

There is an association between the number of co-authors and the number of citations for that article. The analysis included 163 articles by 918 authors. Comparing the number of articles and the number of authors per article generated a normal distribution right-tailed curve (Figure 10). There was a mean of 3 authors per article, with a standard deviation of 3.55 and a mode of 4 authors per article (35 articles had 4 authors). The average number of citations increased slightly with an increase in the number of participating authors, albeit with a low positive correlation of +0.57.

The number of authors who cooperated for one article ranged from 1 author to 23 co-authors. The top 10 most-cited articles had, on average, 7.5 authors per article.

Sud A. et al. (2020), which was published in The Lancet Oncology Journal, had 23 authors, which was the highest number of any paper in this study, and was cited by 152 articles, which was the most citations of all of the included manuscripts [11]. Govindan K. et al. (2014), which was published in The Hematology Journal, came in second place, with 20 participating authors from the Mexican Association of Pediatric Hematology-Oncology [52].

**Figure 11:**
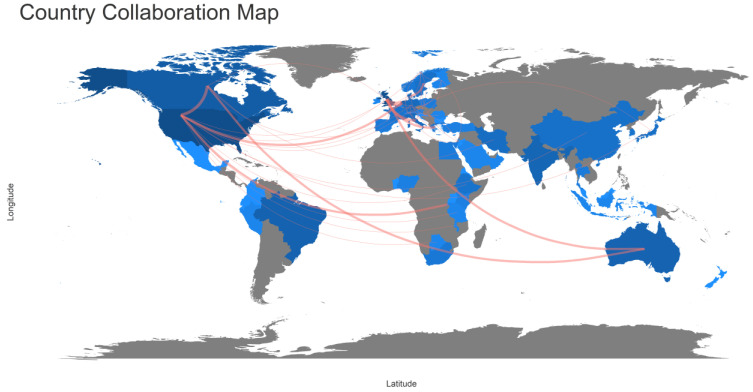
The global network of RPHC research.

Collaboration between scholars does not recognize borders between states and continents, especially given the current communication technology. There is international cooperation between scholars from all of the continents. Figure 11 shows extensive collaboration between North America and Europe, between North America and Australia, between North America and Africa, and between Australia and Europe. The thickness of the arc connecting two countries reflects the number of articles for which collaboration occurred, and the color of the countries reflects their contributions to that field. The United States had a strong collaboration with Canada, Europe, China, Australia, and India. Table 7 shows that 29 of the 163 articles are MCP (Multi countries’ Publications) (18%). The USA, the UK, and China show high contribution and collaboration, with 10%, 27%, and 40% MCP rations, respectively. However, Canada has a high contribution of 18 articles to the RPHC field, all of which are single country publications, with 0% MCP ratio.

## 4. Categorization and Evaluation (Qualitative)

### 4.1. Clustering and Grouping of Articles

This section analyzes selected papers in order to construct a complete view of the recent state-of-the-art studies on RPHC using a modification of the Govindan model [53]. The results clarify the current gaps and discuss future directions for research. The articles were classified and categorized based on the aim of the study, the research paradigm, the disease type, the referral direction, and whether the study was local or international (Table 8).

### 4.2. Categorization of Articles Based on the Research Aim and Primary Objective

#### 4.2.1. Descriptive Analysis Papers

Seventy-seven articles answered questions about referrals and their role in healthcare services, the challenges in making a good referral, and the referral performance. These articles analyzed the referral pattern using both qualitative and quantitative paradigms. They used data from surveys, interviews, debates, or focus groups consisting of leading participants in the referral process, patients, primary caregivers, and specialists. Quantitative methods were used in order to define the problems or the issues that required intervention (Table S2).

This category can be subdivided into the following three main themes:

Referral Decisions. This includes why the general physicians refer the patients to a specialist, the types of specialists to whom the patients are commonly referred, and the referral rate.

Accessibility. This includes the enablers and the barriers to specialty care referral, the waiting time, and the tools that are available to manage the access to care, including triage and waiting lists. The accessibility varies depending on the healthcare systems. Some countries adopt public healthcare systems, e.g., Canada, China, Qatar, and the UK, while others rely more on private healthcare systems, e.g., the USA [1]. This study has examined the variation in specialty referral rates between the USA and the UK systems.

Communication and Coordination. This refers to the quality of the contact between all of the participants in the referral process, the tools that are used for information management, and the coordination between the facilities that is necessary in order to make the referral process function.

After defining these themes, it was important to understand the effect of these factors on the referral performance and the outcomes.

#### 4.2.2. Cause and Effect Papers

Twelve papers were identified that examined the association between referral delays and the mortality rate, or the association between particular components of the referral process and general referral patterns. These manuscripts used the quantitative paradigm in order to answer their central question, and the majority studied the primary referral for cancer (Table S3). It was evident from these studies that an additional class of research is needed in order to focus on developing solutions and optimizing the referral process.

#### 4.2.3. Intervention Papers

Fifty papers were identified that focused on interventions aiming to reduce barriers and to optimize the referral process. Out of these, half of them (25 articles) used modeling prospects, while the other half used non-modeling prospects. Operational research addresses issues in health care by taking advantage of the improvements in computer science and technology. The simulation system helps us to build healthcare models and to detect processes at macro and micro levels. Combined with optimization technology, this type of research can create a comprehensive model for solving problems using optimized solutions. Artificial intelligence (AI) and machine learning (ML) can also automate many of the processes in the referral pathway (Table S4).

The following four types of non-modeling interventions were described: electronic referrals, peer feedback, templates, and electronic health records. Combining different intervention types can improve the content and the appropriateness of the referral. Seven of these papers were systematic reviews. The lack of systematic reviews on operational research assessing models for improving the referral pathway is a gap that should be filled (Table S5).

Finally, after using both modeling and non-modeling techniques to optimize the referral process, it is necessary to create guidelines that outline these interventions and audit the adherence of the healthcare systems. Such guidelines are the scope for the last category of papers.

#### 4.2.4. Quality Management Papers

Twenty-four papers were divided into two main subcategories. The first included studies that aimed to build guidelines for the referral process and to study patient satisfaction. The second aimed to audit the adherence of healthcare facilities to the referral guidelines (Table S6). Reviewing these papers revealed some gaps that could be addressed in the future research on the RPHC (Section 5).

## 5. Research Gaps and Future Directions

### 5.1. Research Gaps

This study revealed a shortage of articles that examined the primary referral process in blood cancer care. Indeed, additional research is needed in order to evaluate the factors that prompt primary care physicians to refer a patient to a hematologist. There was also a lack of research that used operational techniques in order to reduce delays and maximize the quality of the referral process. An ML model using AI could be an innovative tool for reading blood tests and aiding the early diagnosis of blood cancer patients. Alternatively, a simulation model could be developed for cancer patient referrals at macro and micro levels. More in-depth qualitative research on the best practices for collaboration and coordination between government and private healthcare facilities is needed, particularly during a pandemic.

### 5.2. Future Research Directions

**Figure 12:**
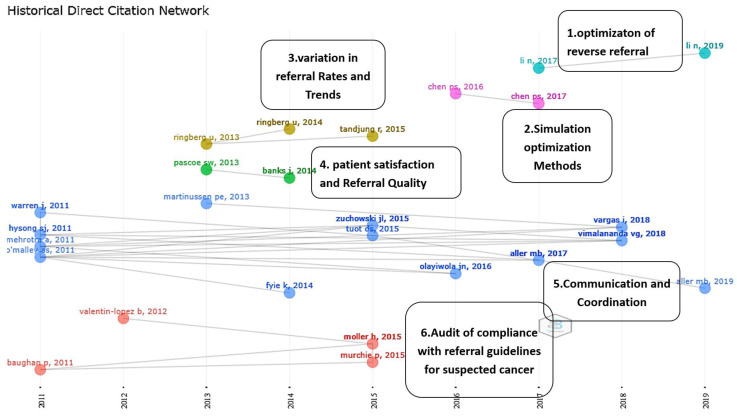
Histography of the future research directions. [3,33,37,40,41,54,55,56,57,58,59,60,61,62,63,64,65,66,67,68,69,70,71,72,73,74].

There are two main directions for future research, operations management, and health quality management. A citations network was created using a histogram, one of which with Biblioshiny graph options, with each node reflecting the most impactful articles on RPHC. The connections from the right to left nodes indicate direct citations, with the horizontal axis representing time. The articles that are represented by the same color share the same research direction. The analysis revealed six future research directions (Figure 12).

#### 5.2.1. Optimization of the Reverse Referral

Reverse referrals appear to represent a new research direction that depends entirely on operations management research. These studies focused on assessing the alliances between the public and the specialized hospitals in China, where reverse referrals are used to balance the referral load and to reduce the waiting times. The medical service cost is the primary variable in the optimization model [41,54]. More research is needed in different health care systems outside of China in order to analyze the validity of these models.

#### 5.2.2. Simulation Optimization Models

Operation management uses simulation and optimization models for solving the referral problems in healthcare. This trend was developed due to innovations in computer programming and the development of information technology, which allow problems to be analyzed using simulations. The outcomes of the referral process can be optimized using this method [55,56]. Other models, such as AI, deep learning (DL), and ML, are also being used to solve problems with the referral process and to open the door for new research possibilities [75].

#### 5.2.3. Variation in Referral Rates and Trends

The variation in the referral rates is studied in order to assess the determinants of referrals, the barriers to referral, and the reasons for referral [57,76]. This research direction can be used as a starting point in order to study the reasons for the difference in referral rates between clinicians, to identify and quantify the determinants of successful referrals based on standardized criteria, and to build referral guidelines based on physician qualifications [58].

#### 5.2.4. Patient Satisfaction and Referral Quality

The core measurement of referral quality is patient satisfaction. This can be analyzed using quality research [59,60], or included as a variable in the referral algorithms that are used in operation research models. Patient involvement in decision making during the referral process is considered to be a patient right.

#### 5.2.5. Communication and Coordination

- A rich research direction consists of many sub-directions. Communication research was previously only concerned with the communication between the general practitioners and the specialists. Later, patient participation in the communication processes became one of the pillars of communication quality [61];
- Electronic referral, E-consultation, and access to the E-health records. The shift from paper to electronic referral has greatly improved the quality of communication. However, this change has had challenges, prompting the development of new studies to facilitate these information management technologies [22,37,62,63];
- The communication breakdown and the methods to address it. Understanding and resolving communication issues, such as insufficient data in referral letters and not receiving a response from specialists, requires the inclusion of the point of view of all of the participants in the referral process through qualitative tools, such as surveys and interviews [64,65];
- The coordination or alliance between the primary care facilities and secondary or tertiary facilities. This can be beneficial to patients, both directly and indirectly; however, this coordination does not come without difficulties [40].

#### 5.2.6. Referral Guidelines and Compliance Audit for Urgent Referral of Suspected Cancer Patients

A quality management research direction starts by improving the performance and building guidelines, then gathering feedback and ensuring compliance with the referral guidelines in order to restart the cycle by understanding the problems and their causes and considering the best tools that could be used to solve them [66,67,68,69].

## 6. Conclusions and Limitations

The bibliometric analysis of the RPHC literature shows an increase in the number of new articles being published between 2010 and 2021 with an increasing rate of citations of these publications. Minimal attention was given to the study of blood cancer, especially concerning the primary referral process for patients with blood cancer versus those with solid tumor types, which is a gap that future studies should address. Scholars in the RPHC field were found to cooperate on articles in the RPHC field, which is likely because of its multidisciplinary nature. A small positive correlation was detected between the number of authors of a particular article and the article’s impact, which likely encouraged additional cooperation. Developed countries were found to cooperate most often with other developed countries, which may change with improvements in communication technology. The keyword analysis showed trends from process orientation to patient orientation by repeating words such as satisfaction and perception.

The content analysis of the articles of interest showed that they could be categorized in several different ways, as follows: the research field, the referral type, the methodology, whether they were clinical or operational studies, and whether they were qualitative or quantitative. The current study categorized the articles based on their aims and objectives. It developed the following four main topics: (i) a descriptive analysis of the definition and an analysis of the referral process, (ii) an analysis of the interventions that are used in order to enhance the referral process, (iii) quality and process guidelines, and (iv) the association between the causes and the effects. The other categorizations included the disease type, the referral type, and the directions.

This review focused solely on the primary referral process and does not provide insights into the problems or the solutions regarding other referral forms. However, most of the studies indicated that the primary referral is the critical process in the pathway. Limiting the search to articles that were published from 2010 to 2021 may mean that some studies with relevant information were not considered in the analysis. However, the systematic reviews that were published during this time frame offer some insight into the older literature. The focus on recent studies increases the applicability of the results for contemporary practice in health systems.

## Figures and Tables

**Table 1:** Keywords used in the search.

Keywords
“E-referral*” OR “referring*” OR “Referral*” OR “Secondary care*” OR “Specialty care*” OR “Provider-provider communications*” OR “Medical transfer*” OR “*Referral” OR “ Diagnosis Delay*” OR “Delay in Diagnosis*” OR “Patient-and-physician matching*” OR “Two-tier healthcare systems*” OR “Referral rate*” OR “Referral payment*” OR “Referral alliance*” OR “Reverse Referral*” OR “mutual referral*” OR “Referral process*” OR “Physician Referral Decision*” OR “e-referral system*” OR “patient-based*” OR “Specialty referral*” OR “Referral system*” OR “specialties*” OR “Referral protocol computerization*” OR “connected healthcare*” OR “online referral*” OR “referral letter*” OR “electronic referral*” OR “waiting to access*” OR “wait list*” OR “Patient-referring mechanism*”
AND
“Hospital quality*” OR “Hospital referral*” OR “Hospital collaboration*” OR “Healthcare*” OR “Patient referral problem*” OR “Hospital*” OR “Hospital collaboration*” OR “Magnetic resonance imaging*” OR “Healthcare systems*” OR “interhospital*” OR “patient referral network*” OR “Healthcare coordination*” OR “Hospital*” OR “integrated healthcare*” OR “Medical services*” OR “Hospital collaboration*” OR “Primary health care*” OR “Specialties*” OR “medical Clinical practice*” OR “Peer review*” OR “Quality of healthcare*” OR “General practice*” OR “Primary care*” OR “Health system*” OR “Intervention*” OR “Secondary care*” OR “Specialty care*” OR “Clinical decision support*” OR “Ambulatory care*” OR “Specialist*” OR “health communication*” OR “health system*” OR “quality of health care*” OR “consultation*” OR “waiting list*” OR “Diagnosis*” OR “health centres*” OR “general medical*” OR “healthcare innovation*” OR “patient centered care*” OR “access to care*” OR “cancer*” OR “oncolog*” OR “tumor*” OR “tumour*” OR “carcinoma*” OR “hematology*” OR “hematologic*” OR “haematology*” OR “hematologist” OR “hematopoietic*” OR “blood cancer*” OR “leukemia*” OR “lymphocyte*” OR “hodgkin lymphoma*” OR “lymphoma*” OR “myeloma*”
AND
“Artificial intelligence*” OR “Data mining*” OR “Decision support systems*” OR “Expert systems*” OR “Machine learning*” OR “Optimization*” OR “Support vector machines*” OR “Heuristic algorithm*” OR “Particle swarm optimization*” OR “Simulation optimization*” OR “Coordination*” OR “Decision making*” OR “Threshold control policy*” OR “bat algorithm*” OR “Decision support system*” OR “Fuzzy multi-criteria decision-making*” OR “Computed tomography*” OR “Collaboration*” OR “Simulation*” OR “queueing theory*” OR “Bayesian inference*” OR “exponential random graph models*” OR “interorganisational networks*” OR “Monte Carlo methods*” OR “statistical models for social networks*” OR “Control agreement framework*” OR “Multi-Fidelity Model*” OR “Pareto Optimization*” OR “Coordination control*” OR “Real-time system*” OR “Heuristic algorithm*” OR “Service development*” OR “Variation*” OR “Service design*” OR “Waiting times*” OR “decision support*” OR “Knowledge modeling*” OR “Referral protocol computerization*” OR “Semantic web bottleneck*” OR “information flow*” OR “queueing*” OR “Process improvement*” OR “Wait times*” OR “Operations Research*” OR “Optimi*” OR “simulat*”

**Table 2:** Number of publications and total citations.

Year	Number of Articles	Cumulative Number of Articles	Total Citations (Google Scholar)	Cumulative Number of Citations	Average Citations per Article	Average Citations per Year
2010	8	8	542	542	67.8	47.1
2011	12	20	994	1536	82.8	94.7
2012	11	31	545	2081	49.6	57.4
2013	11	42	466	2547	42.4	53.0
2014	8	50	316	2863	39.5	42.1
2015	14	64	429	3292	30.6	66
2016	11	75	491	3783	44.6	89.3
2017	15	90	212	3995	14.2	47
2018	23	113	392	4387	17.1	112
2019	19	132	154	4541	8.1	61.5
2020	21	153	230	4771	11	153.3
2021 *	10	163	15	4786	1.5	30

N.B. *: only the first 6 months of 2021 were included in the study.

**Table 3:** Characteristics of prior review studies.

Paper	Area	Scope	Methodology and Structure	Year	Number of Papers
Liddy et al. (2019) [22]	HCSS ^a^	E-consultation effect on RP	Systematic review	2014–2017	43
Delva et al. (2012) [28]	Oncology	Effect of Age on RP	Systematic Review	Until July 2010	31
Pittalis et al. (2019) [27]	Science and Technology	Effect of Income Variation on the RP	Systematic review	2008–2018	14
Rathnayake and Clarke (2021) [17]	HCSS ^a^	Impact of intervention on Waiting time	Systematic review	2014–Jan 2020	9
Lewis et al. (2018) [18]	HCSS ^a^	Effect of intervention on Waiting time	Case studies	Until 2017	–
Villeneuve et al. (2013) [19]	Rheumatology	Effect of intervention on Waiting time	Systematic Review	1985–Nov 2010	47
Mansell et al. (2011) [20]	General and Internal Medicine	Effect of intervention on Waiting time	Systematic Review	Until March 2010	22
Tobin-Schnittger et al. (2018) [23]	General and Internal Medicine	Improving Quality of Referral letter	Hybrid Review	2007–2017	18
Janssen et al. (2020) [21]	General and Internal Medicine	Intervention to promote collaboration	Systematic Review	1960–April 2019	44
Salins et al. (2020) [24]	HCSS ^a^	Palliative care Referral in Cancer	Systematic Review	1990–2019	23
Hui et al. (2016a) [26]	Oncology	Palliative care Referral in Cancer	Delphi	Until 2015	–
Hui et al. (2016b) [25]	Oncology	Palliative care Referral in Cancer	Systematic Review	1947–2015	21
Guevara et al. (2011) [30]	HCSS ^a^	Performance Measures of RP	Systematic Review	Until June 2009	214
Sussman and Baldwin (2010) [31]	Oncology	Referral process in Cancer care	Peer review	Until 2010	–
Greenwood-Lee et al. (2018) [14]	HCSS ^a^	Problem Solution to improve RPHC ^c^	Narrative Review	2005–2014	106
Blank et al. (2014) [13]	General and Internal Medicine	Problem Solution to improve RPHC ^c^	Systematic Review	2000–July 2013	140
Our Study	HCSS ^a^ and OM ^b^	Problem Solution to improve RPHC ^c^	Hybrid Review	2010–June 2021	163

N.B.: ^a^ HCSS: Health care sciences and services. ^b^ O.M: Operation management. ^c^ RPHC: Referral pathway in healthcare.

**Table 4:** Top ten most-cited articles and rank per year.

Rank	Paper Type	Title	Number of Citations	Citations per Year	Rank Citations per Year
1	Article	Variation in number of general practitioner consultations before hospital referral for cancer: findings from the 2010 National Cancer Patient Experience Survey in England [32]	348	34.8	2
2	Article	Dropping the Baton Specialty Referrals in the United States [33]	315	28.6	3
3	Article	Referral and Consultation Communication Between Primary Care and Specialist Physicians Finding Common Ground [3]	282	25.6	4
4	Article	The interface between primary and oncology specialty care: Treatment through survivorship [34]	227	18.9	6
5	Article	Effect of delays in the 2-week-wait cancer referral pathway during the COVID-19 pandemic on cancer survival in the UK: a modelling study [11]	152	76	1
6	Review	Referral criteria for outpatient specialty palliative cancer care: an international consensus [26]	150	25	5
7	Review	A systematic literature review of strategies promoting early referral and reducing delays in the diagnosis and management of inflammatory arthritis [19]	126	14	9
8	Article	Explaining variation in referral from primary to secondary care: a cohort study [35]	104	8.7	*
9	Article	Cancer suspicion in general practice, urgent referral, and time to diagnosis: a population-based GP survey and registry study [36]	98	12.3	*
10	Article	Electronic Consultations to Improve the Primary Care–Specialty Care Interface for Cardiology in the Medically Underserved: A Cluster-Randomized Controlled Trial [37]	90	15	8
*	Review	A Systematic Review of Asynchronous, Provider-to-Provider, Electronic Consultation Services to Improve Access to Specialty Care Available Worldwide [22]	46	15.3	7
*	Review	Referral Criteria for Outpatient Palliative Cancer Care: A Systematic Review [25]	82	13.6	10

N.B. *: Not in the Top 10.

**Table 5:** Top 10 author contributions in RPHC from 2010 to 2021.

Authors	Number of Documents	H-Index	Average Citations per Document	The First Article (2010–2021)
Lyratzopoulos G.	7	5	31	(Lyratzopoulos G. et al., 2012)
Abel G.A.	6	5	29.7	(Abel, G. A. et al., 2012)
Vargas I.	5	3	46.8	(Aller, M. B., et al., 2017)
Hamilton W.	5	3	42	(Banks, J., et al., 2014)
Vazquiz M.L.	4	3	51.3	(Aller, M. B., et al., 2017)
Liddy C.	4	3	41	(Liddy, C., et al., 2017)
Vmalanada V.G.	4	3	36	(Vimalananda, V. G., et al., 2019)
Vedsted P.	4	3	34.8	(Jensen, H. et al., 2014)
Rubin G.	4	3	31.8	(Lyratzopoulos G. et al., 2012)
Mendonca S.C.	4	3	29.3	(Mendonca, S. C. et al., 2016)
Chen P.S.	4	2	21.3	(Chen, P. S., et al., 2016)

**Table 6:** Research area and science category.

Web of Science Categories	Articles	Percentage
Health Care Sciences and Services	57	35%
Medicine, General, and Internal	40	25%
Primary Health Care	29	18%
Medical Informatics	21	13%
Health Policy and Services	20	12%
Oncology	19	12%
Computer Science	11	7%
Public, Environmental, and Occupational Health	11	7%
Management	7	4%
Operations Research	6	4%
Engineering	6	4%
Hematology	6	4%
Multidisciplinary Sciences	5	3%
Interdisciplinary Applications	4	2%
Biomedical	3	3%
Pediatrics	3	1%
Nursing	3	1%
Artificial Intelligence	2	1%
Radiology	2	1%
Rehabilitation	2	1%
Nuclear Medicine and Medical Imaging	2	1%
Others (only 1 Article)	12	8%

**Table 7:** Countries’ publication showing single country publications (SCP), multiple countries publications (MCP), and MCP ratio of each country.

Country	Articles	Frequency	SCP	MCP	MCP_Ratio
USA	50	28%	45	5	10%
UK	33	19%	24	9	27%
Canada	18	11%	18	0	0%
Australia	10	6%	9	1	10%
China	10	6%	6	4	40%
The Netherlands	8	5%	5	3	38%
Norway	7	4%	7	0	0%
Spain	5	3%	4	1	20%
Ireland	4	3%	4	0	0%
Denmark	3	2%	3	0	0%
Brazil	2	2%	1	1	50%
France	2	2%	2	0	0%
Italy	2	2%	1	1	50%
Switzerland	2	2%	2	0	0%
India	2	1%	1	1	50%
Bangladesh	1	1%	0	1	100%
Germany	1	1%	1	0	0%
Japan	1	1%	1	0	0%
Mexico	1	1%	0	1	100%
Morocco	1	1%	0	1	100%
Total	163	100%	134	29	18%

**Table 8:** Categorization and classification of the articles.

Main Classes	Aim of the Research	Research Paradigm	Type of Disease	Research Region	Referral Direction	Data Collection
Main class 1	Descriptive	Qualitative	Cancer	Localized	Primary	Primary data
Main class 2	Cause effect	Quantitative	Others	Globalized	Cross Palliative	Secondary data
Main class 3	Interventions	Mixed	General	General	Reversed	Mixed
Main class 4	Quality	Modeling	-	-	General	-

## Data Availability

Data is available in the supplementary material and upon reasonable request to corresponding author.

## Supplementary Materials

The following supporting information can be downloaded at: https://www.mdpi.com/article/10.3390/ijerph192416952/s1, Table S1: Journal contribution and its impact factor; Table S2: Descriptive analysis papers on RPHC, arranged in descending order using citations per year; Table S3:Papers studying the cause and effect and association analysis arranged in descending using citations per year; Table S4: Papers of interventions to solve referral problems using operational management models; Table S5: Papers of interventions to solve referral problems using management review arranged in descending using citations per year; Table S6: Articles focusing on the quality management of the referral pathway ranked in descending order by the article citation number per year. References [77,78,79,80,81,82,83,84,85,86,87,88,89,90,91,92,93,94,95,96,97,98,99,100,101,102,103,104,105,106,107,108,109,110,111,112,113,114,115,116,117,118,119,120,121,122,123,124,125,126,127,128,129,130,131,132,133,134,135,136,137,138,139,140,141,142,143,144,145,146,147,148,149,150,151,152,153,154,155,156,157,158,159,160,161,162,163,164,165,166,167,168,169,170,171,172,173,174,175,176,177,178,179,180,181] are cited in the supplementary files.

## Abbreviations

AI: Artificial intelligence; AU: Authors; CR: Cited references; DL: Deep learning; GP: General practitioner; HCSS: Healthcare Science and Services; H-index: Hirsch index; ML: Machine learning; NICE: National Institute for clinical excellence; OM: Operations management; PCP: Primary care physician; RP: Referral process; RPHC: Referral process in healthcare; WHO: World health organization.
