# Supplementary material for: A Bibliometric Analysis and Visualization of Decision Support Systems for Healthcare Referral Strategies
Source: Int J Environ Res Public Health. 2022 Dec 16;19(24):16952. doi: 10.3390/ijerph192416952 (PMC9778793; doi:10.3390/ijerph192416952)
Supplement: Supplementary file 1 [file ijerph-19-16952-s001.zip › ijerph-1879526-Supplementary.pdf]

# Supplementary Table S1

Table S1 shows the contributions of the journals using the number of articles and their impacts using Eigenfactor. The Eigenfactor came out of the Metrics Eigenfactor Project in 2008, a bibliometric research project conducted by Professor Carl Bergstrom and his laboratory at the University of Washington. All data come from checking the journal impact using IF Clarivate Analytics JCRI 2021.

Table S1: Journal contribution and its impact

| **Source Title (Journal)** | **Articles** | **Rank** | **Total Cites** | **Journal Impact Factor** | **Eigenfactor Score** | **Quartile** |
|---|---|---|---|---|---|---|
| BMC HEALTH SERVICES RESEARCH | 15 | 5755 | 25,956 | 2.655 | 0.04173 | Q2 |
| BMC FAMILY PRACTICE | 11 | 6172 | 5,727 | 2.497 | 0.00809 | Q2 |
| BRITISH JOURNAL OF GENERAL PRACTICE | 10 | 1677 | 8,303 | 5.386 | 0.00930 | Q1 |
| INTERNATIONAL JOURNAL OF MEDICAL INFORMATICS | 5 | 2991 | 7,651 | 4.046 | 0.01044 | Q1 |
| PLOS ONE | 5 | 4371 | 857,723 | 3.240 | 1.08115 | Q2 |
| OMEGA-INTERNATIONAL JOURNAL OF MANAGEMENT SCIENCE | 4 | 933 | 12,730 | 7.084 | 0.01084 | Q1 |
| LANCET ONCOLOGY | 3 | 34 | 72,804 | 41.316 | 0.13853 | Q1 |
| Journal of the National Cancer Institute - Monographs | 3 | 264 | 42,005 | 13.506 | 0.03826 | Q1 |
| JOURNAL OF GENERAL INTERNAL MEDICINE | 3 | 1883 | 26,727 | 5.128 | 0.02895 | Q1 |
| CURRENT ONCOLOGY | 3 | 3537 | 3,567 | 3.677 | 0.00567 | Q2 |
| BMC PALLIATIVE CARE | 3 | 4388 | 2,800 | 3.234 | 0.00523 | Q2 |
| MEDICAL DECISION MAKING | 3 | 5940 | 6,391 | 2.583 | 0.00724 | Q2 |
| APPLIED CLINICAL INFORMATICS | 3 | 6687 | 1,481 | 2.342 | 0.00290 | Q3 |
| BRITISH JOURNAL OF CANCER | 2 | 784 | 54,924 | 7.640 | 0.04207 | Q1 |
| BMJ QUALITY & SAFETY | 2 | 945 | 7,335 | 7.035 | 0.01436 | Q1 |
| ANNALS OF FAMILY MEDICINE | 2 | 1848 | 6,770 | 5.166 | 0.00928 | Q1 |
| SOCIAL SCIENCE & MEDICINE | 2 | 2296 | 57,968 | 4.634 | 0.05213 | Q1 |
| JOURNAL OF MEDICAL SYSTEMS | 2 | 2442 | 8,017 | 4.460 | 0.00950 | Q1 |
| BMJ SUPPORTIVE & PALLIATIVE CARE | 2 | 3726 | 2,039 | 3.568 | 0.00418 | Q2 |
| HEALTH POLICY AND PLANNING | 2 | 4143 | 6,543 | 3.344 | 0.01063 | Q2 |
| CANADIAN FAMILY PHYSICIAN | 2 | 4298 | 4,641 | 3.275 | 0.00535 | Q2 |
| SCANDINAVIAN JOURNAL OF PUBLIC HEALTH | 2 | 4848 | 7,088 | 3.021 | 0.00546 | Q2 |
| JMIR MEDICAL INFORMATICS | 2 | 4998 | 1,343 | 2.955 | 0.00369 | Q2 |
| BMJ OPEN | 2 | 5652 | 55,068 | 2.692 | 0.14519 | Q2 |
| EUROPEAN JOURNAL OF CANCER CARE | 2 | 6089 | 4,547 | 2.520 | 0.00659 | Q2 |
| INFORMATICS FOR HEALTH & SOCIAL CARE | 2 | 6381 | 511 | 2.439 | 0.00083 | Q2 |
| JOURNAL OF EVALUATION IN CLINICAL PRACTICE | 2 | 6398 | 5,408 | 2.431 | 0.00534 | Q2 |
| JOURNAL OF MULTIDISCIPLINARY HEALTHCARE | 2 | 6485 | 1,737 | 2.404 | 0.00319 | Q2 |
| FAMILY PRACTICE | 2 | 6906 | 5,336 | 2.267 | 0.00446 | Q3 |
| PRIMARY HEALTHCARE RESEARCH AND DEVELOPMENT | 2 | 9514 | 1,013 | 1.458 | 0.00194 | Q3 |
| Others (Only 1 Article) | 58 | | | | | Q1-Q2-Q3 |

# Supplementary Table S2

Table S2: Descriptive analysis papers on RPHC, arranged in descending order using citations per year

| Authors / GC | Article Title | where | Disease | Paradigm/Methods/Sample size | Type of Referral | conclusion |
|---|---|---|---|---|---|---|
| **Lyratzopoulos et al. (2012)/348** [32] | Variation in number of general practitioner consultations before hospital referral for cancer: findings from the 2010 National Cancer Patient Experience Survey in England | England | Cancer | Quantitative/Findings from the 2010 National Cancer Patient Experience Survey in England/41 299 patients with 24 different cancers | Primary | Prioritize and stratify early diagnosis initiatives and research, focusing on cancer patients and sociodemographic characteristics with the most considerable potential for improvement. |
| **Mehrotra et al. (2011)/315** [33] | Dropping the Baton: Specialty Referrals in the United States | USA | General | Qualitative/Narrative Review | Primary | There are breakdowns and inefficiencies in all components of the specialty referral process. Despite many promising mechanisms to improve the referral process, |
| **O'Malley; Reschovsky (2011)/282** [3] | Referral and Consultation Communication Between Primary Care and Specialist Physicians Finding Common Ground | General | General | Mixed/Physician Survey / 4720 physicians provided at least 20 hours per week of direct patient care. | Primary | This modifiable practice supports communication between PCPs and specialists to help inform how resources focus on improving care coordination. |
| **Hui et al. (2016a)/150** [26] | Referral criteria for outpatient specialty palliative cancer care: an international consensus | General | Cancer | The Qualitative/Delphi panel consisted of international experts | Cross/ Palliative | The international panel agreed on 11 primaries and 36 minor criteria for outpatient palliative care referral for patients with advanced cancer treated at secondary or tertiary care hospitals. |
| **Grunfeld; Earle (2010)/227** [34] | The interface between primary and oncology specialty care: Treatment through survivorship | General | Cancer | Qualitative | Primary | Empowering patients to take responsibility for their care and ensuring clarity around where responsibility for cancer surveillance |
| **Hui et al. (2016b)/82** [25] | Referral Criteria for Outpatient Palliative Cancer Care: A Systematic Review | General | Cancer | Mixed/Systematic review | Cross/ Palliative | This systematic review identified 20 criteria, including six recurrent palliative care referral themes. It highlights the significant heterogeneity regarding the timing and process for the referral. |

| | | | | | | |
|---|---|---|---|---|---|---|
| **Jensen et al. (2014)/98** [36] | Cancer suspicion in general practice, urgent referral and time to diagnosis: a population-based GP survey and registry study | General | Cancer | Qualitative/Population-based cross-sectional study/5,581 questionnaires | Primary | GPs suspect cancer more often than they initiate a CPP, and patients were less likely to be referred to a CPP when their symptoms were not interpreted as alarm symptoms of cancer. Still, the GP's symptom interpretation was approximately twice as strong an indicator of a more extended diagnostic break. |
| **Jaakkimainen et al. (2014)/81** [4] | Waiting to see the specialist: patient and provider characteristics of wait times from primary to specialty care | Canada | General | Quantitative/Statistical | Primary | Actual waiting times for a referral from a Family Physician to a specialist physician are longer than those reported by physician surveys |
| **Zuchowski et al. (2015)/67** [73] | Challenges in Referral Communication Between VHA Primary Care and Specialty Care | General | General | Qualitative/Survey and Interviews | Primary | Improvement in EMR systems is needed, with more flexibility for communicating special requests. Building relationships between PCPs and specialists may also facilitate referral communication. |
| **Vargas et al. (2018)/38** [64] | Understanding communication breakdown in the outpatient referral process in Latin America: a cross-sectional study on the use of clinical correspondence in public healthcare networks of six countries | Latin America | General | Qualitative/cross-sectional study | Primary | This study highlights significant problems in using referrals and replies to letters in the public networks studied in six Latin American countries: the infrequent or late receipt of the response to letters, and in some countries also, referral letters and the low quality of data recording in them. |
| **Easley et al. (2017)/45** [77] | The role of family physicians in cancer care: perspectives of primary and specialty care providers | Canada | Cancer | Qualitative/Telephone interviews with 58 primary and cancer specialists | Primary | We need better communication, more collaboration, and further education to enhance family physician's role in the care of cancer patients. |
| **Mendonca et al.(2016a)/52** [78] | Pre-referral general practitioner consultations and subsequent experience of cancer care: evidence from the English Cancer Patient Experience Survey | UK | Cancer | Mixed/Survey | Primary | provided large-scale evidence from a real-world setting suggesting that less prompt referral for specialist assessment after symptomatic presentation negatively affects subsequent cancer care experience. |
| **McBride et al. (2010)/104** | Explaining variation in referral from primary to | UK | General | Quantitative/Statistical/5492 patients with postmenopausal bleeding, 23 121 | Primary | Referral inequality is associated with socioeconomic conditions in |

| | | | | | | |
|---|---|---|---|---|---|---|
| [35] | secondary care: a cohort study | | | with hip pain, and 101 212 with dyspepsia from 326 general practices | | the absence of explicit guidance. In comparison, disparities with age were evident for all states. |
| **McCaughan et al. (2018)/30** [79] | Palliative care specialists' perceptions concerning referral of haematology patients to their services: findings from a qualitative study | UK | Blood Cancer | Qualitative/semi-structured interviews were conducted with 20 SPC doctors& nurses | Cross/ Palliative | Numerous factors were found to influence the likelihood of referral, some related to the organization and delivery of SPC services, so they were amenable to change. Others were associated with the complex and unique characteristics and pathways of hematological cancers |
| **Hysong et al. (2011)/82** [62] | Towards successful coordination of electronic health record-based referrals: a qualitative analysis | General | General | Qualitative/Interviews & six focus groups | Primary | Electronic referrals remain subject to coordination disruption. However, information transfer between PCPs and sub-specialists is facilitated. Before implementing an EHR to facilitate referrals, clear referral policies must be in place. |
| **Rotar et al. (2018)/26** [80] | Shared decision-making between patient and GP about referrals from primary care: Does gatekeeping make a difference? | 31 European countries and Australia, Canada, and New Zealand. | General | Qualitative/GPs survey data from the cross-sectional QUALICOPC study | Primary | These results will aid clinicians in having an overall view of international practice. These mixed results may urge policymakers to 1) reconsider the existing primary care mechanisms and 2) rethink the role of the GP as a "gatekeeper" as a guide rather than as a coordinator. |
| **Liss et al. (2011)/69** [81] | Patient-Reported Care Coordination: Associations with Primary Care Continuity and Specialty Care Use | General | General | Qualitative/Cross-sectional study/Survey | Primary | High use of specialty care may strain the ability of primary care clinicians to coordinate care effectively |
| **Aller et al. (2017)/28** [40] | Doctors' opinion on the contribution of coordination mechanisms to improving clinical coordination between primary and outpatient secondary care in the Catalan national health system | Catalonia | General | Qualitative /primary care (n = 26) and secondary care (n = 24) doctors. | General | Mechanisms that allow doctors to exchange information and communicate are enhanced by focusing on adequate working conditions, mechanism design promoting mutual knowledge and positive attitudes towards collaboration. |

| | | | | | | |
|---|---|---|---|---|---|---|
| **Scaioli et al. (2020)/11** [82] | Communication between general practitioners and medical specialists in the referral process: a cross-sectional survey in 34 countries | 34 countries | General | Qualitative/A cross-sectional survey in 34 countries/7183 GPs from 34 countries were surveyed | Primary | Considerable differences in communication between GPs and medical specialists could be explained by the characteristics of the country, and the primary care practice |
| **Ringberg et al. (2014)/44** [70] | Examining the variation in GPs' referral practice: a cross-sectional study of GPs' reasons for referral | General | General | Quantitative/Cross-sectional study | Primary | Reasons for variations in the Referral between Norwegian male and female GPs and between GPs with high and low referral rates reflect difficulties in handling professional uncertainty. |
| **Pidala et al. (2013)/49** [83] | Practice variation in physician referral for allogeneic hematopoietic cell transplantation | General | Blood Cancer | Mixed/statistical analysis/113 physicians semi-structured interviewing and focus group work conducted with HCT physicians | Cross/ Cell Transplantation | Several educational and intervention means are needed to reduce disparate access to HCT. With appropriate access to HCT consultation, patients share to make informed decisions about their therapy. |
| **Wright; Forbes (2017)/27** [84] | Hematologists' perceptions of palliative care and specialist palliative care Referral: a qualitative study | General | Blood Cancer | Qualitative study/face-to-face interviews with a purposive sample of eight trainee and consultant hematologists | Cross/ Palliative | Barriers to collaboration between hematology and SPC services and opportunities to improve interdisciplinary work have been identified as suggestions for an explicit definition of the role of SPC. |
| **Mohr et al. (2019)/16** [85] | Organizational Coordination and Patient Experiences of Specialty Care Integration | General | General | Qualitative/Cross-sectional surveys of patients and primary care providers (PCPs)/3183 patients matched to 233 PCPs | Primary | Coordination between primary care and specialty care services should improve patient experiences of care coordination, care efficiency, and effectiveness. |
| **Martinussen (2013)/42** [61] | Referral quality and the cooperation between hospital physicians and general practice: The role of physician and primary care factors | Norwegian | General | Qualitative/Randomly selected from the records of the Norwegian Medical Association; 1298 questionnaires | Primary | Factors affecting coordination of care. And attempt to address macro and partial barriers to obtaining quality referral care. |
| **Ozturk et al. (2015)/32** [86] | Delay in Diagnosis of Testicular Cancer; A Need for Awareness Programs | General | Testicular Cancer | Qualitative/Questionnaire/Sixty men diagnosed with TC | Primary | The risk variables for longer patient delay were an embarrassment and lower education. An essential risk variable in GPs was 'misdiagnosis.' |
| **Pittalis et al. (2019)/13** | Surgical referral systems in low- and middle-income | Low- and Middle- | Surgical Care | Qualitative/Review/14 studies | Primary | Lack of standard approach to assessing the functionality and |

| | | | | | | |
|---|---|---|---|---|---|---|
| [27] | countries: A review of the evidence | income countries | | | | effectiveness of referral systems in surgery. |
| **Ezeonwu (2018)/17** [8] | Specialty-care access for community health clinic patients: processes and barriers | USA | General | Qualitative descriptive study/semi-structured interviews/37 referral coordinators | Primary | Effective policies must be pursued to Guarantee access to specialty care. |
| | | | | | | |
| **Patel et al. (2018)/16** [87] | Closing the Referral Loop: An Analysis of Primary Care Referrals to Specialists in a Large Health System | General | General | Quantitative/103,737 appointment scheduling attempts | Primary | Strategies to improve scheduling, decrease clinic variation, and improve patient access will likely improve rates of closing the referral loop. |
| **Neimanis et al. (2017)/20** [88] | Referral processes and wait times in primary care | General | General | Quantitative | Primary | Patient and physician frustration is heightened, and more office time and energy are expended when no referral acknowledgment is received within seven weeks. |
| **Mendonca et al. (2016b)/23** [89] | Pre-referral GP consultations in patients subsequently diagnosed with rarer cancers: a study of patient-reported data | General | Rarer Cancer | Quantitative/Survey | Primary | the development of new diagnostic interventions and 'safety netting approaches are required for Patients with rarer cancers |
| **Howell et al. (2015)/26** [90] | Variations in specialist palliative care referrals: findings from a population-based patient cohort of acute myeloid leukaemia, diffuse large B-cell lymphoma, and myeloma | General | Blood Cancer | Quantitative/cohort, 323 patients were diagnosed with acute myeloid leukaemia, diffuse large B-cell lymphoma, or myeloma between May 2005 and April 2008 and died before April 2010. | Cross | Greater integration between hematology and SPC services than previously reported. Even with variations in diagnosis, emphasizing the importance of examining diseases individually |
| **Corbett et al. (2013)/33** [91] | Palliative Care and Hematological Malignancies: Increased Referrals at a Comprehensive Cancer Centre | General | Blood Cancer | Quantitative/Statistical Analysis | Cross | Over recent years, the collaboration between hematology and palliative care has increased referral numbers, with potentially positive results for patients. |
| **Price et al. (2020)/7** [92] | Trends in time to cancer diagnosis around the period of changing national guidance on referral of symptomatic patients: A serial cross- | UK | Cancer | Quantitative/A serial cross-sectional study/Clinical Practice Research Datalink records 83935 case | Primary | Despite improvements for colorectal cancer, the scope remains to reduce diagnostic intervals for most cancers. |

| | | | | | | |
|---|---|---|---|---|---|---|
| | sectional study using UK electronic healthcare records from 2006-17 | | | | | |
| **Aller et al. (2019)/10** [74] | Doctors' opinions on clinical coordination between primary and secondary care in the Catalan healthcare system | Catalonia | General | Qualitative/Semi-structured interviews | Primary | The main barriers to coordination are the lack of time for coordination, regulations variations, and unshared prescribing incentives. |
| **Nekhlyudov; Latosinsky (2010)/37** [93] | The interface of primary and oncology specialty care: From symptoms to diagnosis | General | Cancer | Qualitative | Primary | The phase in the cancer care Avoid unnecessary testing, and referrals are needed. Adequate communication between primary care providers, specialists, and patients. Appropriate wait times that balance better psychological outcomes with lower system costs |
| **Tzartzas et al. (2019)/9** [94] | General practitioners referring patients to specialists in tertiary healthcare: a qualitative study | Switzerland | General | Qualitative/Thematic content analysis | Primary | Referral Decision is influenced by many contextual, personal, and clinical factors that shape the decision-making process. |
| **Banks et al. (2014)/23** [60] | Decision making and referral from primary care for possible lung and colorectal cancer: a qualitative study of patients' experiences | General | Lung& Colorectal Cancer | Qualitative/Interviews/34 patient interviews | Primary | Involving the patients when referring them to investigate symptoms suspicious of cancer encourages more open dialogue between GPs and the patients around cancer. |
| **Fyie et al. (2014)/23** [72] | Evaluating the primary-to-specialist referral system for elective hip and knee arthroplasty | General | Orthopedic care | Qualitative/Mixed-methods retrospective study with semi-structured interviews | Primary | the variation in referral processing led to increased waiting times for patients. Improving referral processes would improve patient access to these specialist referrals |
| **Guevara et al. (2011)/28** [29] | Performance measures of the specialty referral process: a systematic review of the literature | General | General | Mixed/**systematic Review**/214 papers containing 244 measures | Primary | Build a reference for the Performance measures |
| **Esteve-Matali et al. (2020)/5** [95] | Do primary and secondary care doctors have different experiences and perceptions of cross-level clinical coordination? Results of a cross-sectional | Spain | General | Qualitative/ Cross-sectional study /self-administered questionnaire COORDENA-CAT. | All | The COORDENA questionnaire allows us to identify fields for improvement in clinical coordination. |

| | | | | | | |
|---|---|---|---|---|---|---|
| | study in the Catalan National Health System (Spain) | | | | | |
| **Pascoe et al. (2013)/22** [59] | Patients' experiences of referral for colorectal cancer | Australia | Colorectal Cancer | Qualitative/interviews /29 patients | Primary | Patients' relationships with their GP significantly affect care coordination and psychosocial care. They improve patient outcomes by decreasing delays and improving satisfaction, perception of choice, and shared decision-making. |
| **Mendonca et al. (2019)/7** [96] | Associations between general practice characteristics with the use of urgent referrals for suspected cancer and endoscopies: a cross-sectional ecological study | England | Cancer | Quantitative/cross-sectional ecological study | Primary | Practices with younger GPs and training practices generally had higher urgent referrals for suspected cancer, whereas the opposite was true for single-handed practices. |
| **Tandjung et al. (2015)/16** [57] | Referral rates in Swiss primary care with a particular emphasis on reasons for encounter | Swiss | General | Quantitative/Cross-sectional study/60 PCPs | Primary | 94.3% of all issues were solved in primary care, reflecting the crucial role of PCPs as healthcare coordinators. |
| **Kankesan et al. (2013)/20** [97] | Factors associated with referral to medical oncology and subsequent use of adjuvant chemotherapy for non-small-cell lung cancer: a population-based study | General | Lung Cancer | Quantitative/Population-based study/Retrospective cohort study/3354 cases | Primary | The initial decision to refer to medical oncology is associated with the age and stage of the disease. Those factors have an even more significant effect on the decision to overreact. |
| **Liddy et al. (2017)/11** [98] | Primary care physician referral patterns in Ontario, Canada: a descriptive analysis of self-reported referral data | Canada | General | Quantitative/Prospective Study/ primary care providers (PCP) from 20 clinics | Primary | Gastroenterology received the largest share of referrals. |
| **Juliani et al. (2017)/11** [99] | Brazilian Specialists' Perspectives on the Patient Referral Process | Brazil | General | Qualitative research with Phenomenology builds knowledge by exploring people's daily life experiences | Primary | Lack of sufficient resources is a significant problem within HBUs (Health Basic Units) that creates a domino effect throughout the SUS (citizen's rights in Brazil). |
| **Janssen et al. (2020)/4** [21] | Competencies to promote collaboration between primary and secondary | General | General | Qualitative/Thematic analysis approach/44 articles | All | The information collected in this review can support clinicians to enhance and learning collaboration in daily practice and can be used in |

| | | | | | | |
|---|---|---|---|---|---|---|
| | care doctors: an integrative review | | | | | educational programs at all stages of medical education. |
| **Prang et al. (2018)/8** [100] | The use of public performance reporting by general practitioners: a study of perceptions and referral behaviors | General | General | Qualitative /Semi-structured interviews/40 GPs | Primary | The indicators require further development before GPs perceive them as valid, credible, and valuable for informing their referral practices. |
| **Thorsen et al. (2016)/12** [101] | Typologies in GPs' referral practice | General | General | Mixed/Observational cross-sectional study using data from 128 Norwegian GPs /691 referrals | Primary | Training collaboration with patients and hospital counselors may enhance self-reflection on competencies and increase confidence levels at referral. |
| **Abel et al. (2012)/18** [39] | Referrals for suspected hematologic malignancy: A survey of primary care physicians | Massachusetts | Blood Cancer | Mixed/Survey/190 Massachusetts PCPs | Primary | Despite the absence of specific guidelines for screening and surveillance, the overall referral system for suspected hematologic malignancies functions relatively well |
| **Militello et al. (2018)/7** [102] | Hidden complexities in information flow between primary and specialty care clinics | General | General | Qualitative/Using interviews, observations, and document review | Primary | The Computerized Patient Record System (CPRS) appears to support critical communication about consultations and follow-. |
| **Savoy et al. (2019)/5** [103] | Cognitive requirements for primary care providers during the referral process: Information needed from and interactions with an electronic health record system | General | General | Qualitative/interview/62 PCPs | Primary | to adequately support the communication, information exchange, or care coordination related to the referral process. This study provides an essential foundation for developing patient-centered displays that support PCPs' decision-making process and |
| **Tandjung et al. (2017)/8** [76] | Referral determinants in Swiss primary care with a special focus on managed care | Swiss | General | Quantitative/statistical/cross-sectional study with 90 PCPs | Primary | Found a nonlinear relationship between referral rate and age with lower odds of referral in older patients. This study emphasizes the central role of primary care providers as care coordinators. |
| **Gulla et al. (2017)/8** [104] | User Requirements for a Chronic Kidney Disease Clinical Decision Support Tool to Promote Timely Referral | General | Kidney Disease | Qualitative/ Interviews | Primary | These 21 user requirements can design an intuitive and usable CDS system with the attributes necessary to promote timely referrals. |

| | | | | | | |
|---|---|---|---|---|---|---|
| **Davies et al. (2011)/17** [105] | What do we know about the referral process? | General | General | Qualitative/Debate | Primary | Given that this process generates 90% of NHS costs, this ignorance is no longer accepted |
| **Salins et al. (2020)/3** [24] | How views of oncologists and haematologists impacts palliative care referral: a systematic review | General | Cancer | Qualitative/Narrative Review/23 studies | Cross | an integrated model of care between Oncologists and hematologists, changing the name of palliative care, and augmenting palliative care resources might facilitate a referral. |
| **Burton et al. (2017)/7** [106] | Distinguishing variation in referral accuracy from referral threshold: analysis of a national dataset of referrals for suspected cancer | England | Cancer | Quantitative/statistical/5479 general practitioner (GP) practices with data relating to more than 50 cancer cases diagnosed over five years. | Primary | Changing referral thresholds without increasing accuracy will result in modest effects on detection rates and a significant increase in demand for diagnostic services. |
| **Kirkpatrick et al. (2010)/16** [107] | Referral Patterns and Adjuvant Chemotherapy Use in Patients with Stage II Colon Cancer | General | Colon Cancer | Quantitative/Statistical | Primary | Referral patterns afford an understanding of recurrence risk and optimize the patient selection for adjuvant chemotherapy. |
| **Dunlea; Lenert (2015)/9** [108] | Understanding Patients' Preferences for Referrals to Specialists for an Asymptomatic Condition | USA | General | Qualitative/Survey and Interviews | Primary | Referral of severe but asymptomatic condition costs are significant to patients; however, they value better communication and variability in specialist preferences. |
| **Gaytan-Morales et al. (2019)/3** [52] | B-cell NHL, early referral and supportive care problems in a developing country | Mexico | B-cell NHL | Quantitative/Statistical/166 pediatric patients were diagnosed with B-cell NHL at participating institutions. | Primary | urgent need for an academic collaboration to create comprehensive strategies to improve pediatric cancer care, especially in diseases with expected excellent prognoses such as B-NHL. |
| **Hirsch et al. (2012)/10** [109] | Referral from primary to secondary care in Germany: Developing a taxonomy based on cluster analysis | Germany | General | Model/Taxonomy/k-means cluster analysis/3,988 referrals by 29 German general practitioners (GPs). | Primary | Taxonomy of referrals that optimize the allocation of resources within the German health system. Improvement of the training of primary care physicians and giving them more competencies in routine care. |
| **Coyle et al. (2011)/7** [110] | Who goes where? A prospective study of referral patterns within a newly established primary care team | Ireland | General | Quantitative/Questionnaires/Prospective Survey | Primary | Referral rates of GPs and their Pattern provide valuable data for future health service planning and health resource allocation. |

| | | | | | | |
|---|---|---|---|---|---|---|
| **Pedersen; Vedsted (2015)/4** [111] | General practitioners' anticipated risk of cancer at referral and their attitude to risk-taking, and their role as gatekeeper | Denmark | Cancer | Qualitative/Questionnaire | Primary | A focus on tolerance for ambiguity should be included in the education of general practitioners. To optimize their Decisions When referring for suspected cancer. |
| **Haste et al. (2020)/1** [112] | Patient experiences of the urgent cancer referral pathway-Can the NHS do better? Semi-structured interviews with patients with upper gastrointestinal cancer | Cumbria Region of England | GIT Cancer | Qualitative/Semi-structured interviews/Twenty patients | Primary | Patient-centered support shared decision-making needs to be designed for future cancer pathways |
| **Vimalananda et al. (2020)/1** [113] | Development and psychometric assessment of a survey to measure specialty care coordination as experienced by primary care providers | General | General | Qualitative/Survey | Reversed | Quality improvement Survey to evaluate care coordination as experienced by either or both participants. |
| **Majhail; Jagasia (2014)/4** [114] | Referral to Transplant Center for Hematopoietic Cell Transplantation | General | Blood Cancer | Qualitative/Model/Descriptive | Cross/ | care models for timely referral to a transplant center and transition from the transplant center back to the referring physician. |
| **Scaioli et al. (2019)/1** [115] | Patients' perception of communication at the interface between primary and secondary care: a cross-sectional survey in 34 countries | 34 countries | General | Qualitative/Cross-sectional survey in 34 countries/Questionnaire/61,931 patients | Primary | Policies stimulating personal doctor arrangements could potentially enhance the continuity of care between primary and secondary care. |
| **Ahmed et al. (2019)/1** [116] | Electronic patient agenda forms: comparing agreement between the reason for specialty consultation reported by referring providers and patients | General | General | Qualitative | Primary | There is a poor agreement between referring providers' reason for GI referral and patients' understanding of why they visit the clinic. |
| **Potappel et al. (2019)/0** [117] | To what degree do patients actively choose their healthcare provider at the point of referral by their GP? A video observation study | Germany | General | Mixed/Videotaped /Statistical analysis/117 of these consultations, with 28 GPs | Primary | half of the patients have some input in their choice of healthcare. Provider at the point of referral by the GP. |

| | | | | | | |
|---|---|---|---|---|---|---|
| **An et al. (2018)/33**<br><br>[118] | Analysis of the U.S. patient referral network | USA | General | Quantitative/algorithms and methods from graph theory, network science, and statistics | All | To improve the referral network in terms of healthcare system efficiency and physician workload by understanding aspects of the network for the most well-performing cases. |
| **Francetic et al. (2021)/0**<br><br>[119] | A network analysis of patient referrals in two district health systems in Tanzania | Tanzania | childhood illnesses and non-communicable diseases | Qualitative/Quality improvement/Network analysis (or social network analysis) | All | Demonstrate the possibility of using network analysis to evaluate patient referrals |
| **Sussman; Baldwin.**<br><br>**(2010)/73**<br><br>[31] | The interface of primary and oncology specialty care: From diagnosis through primary treatment | General | Cancer | Qualitative | Primary | Communications barriers and the interface between the primary and secondary levels of care |
| **Burton et al. (2020)/8**<br><br>[120] | Contribution of primary care organization and specialist care provider to variation in G.P. referrals for suspected cancer: an ecological analysis of national data | General | Cancer | Quantitative/Ecological Analysis of National Data | Primary | Efforts to reduce suspected cancer referrals vary to focus on individual practices, local diagnostic and specialist service provision, and culture. |
| **Liddy et al. (2020)/3**<br><br>[121] | How long are Canadians waiting to access specialty care? A retrospective study from a primary care perspective | Canada | General | Quantitative/Retrospective study | Primary | This research provides decision-makers with an essential context for developing programs and policies to address the primarily overlooked waiting time continuum from referral to final appointment time with a specialist. |
| **Barth et al. (2020)/4**<br><br>[122] | First Referral to an integrated one-palliative care program: a retrospective analysis of its timing | General | Cancer | Quantitative/Statistical/n = 416 patients (deemed incurable) | Cross | integrated onco-palliative care program (IOPC)is an effective organization to enable early integration of P.C. and decrease the aggressiveness of care near the end of life. |
| **Harris et al. (2012)/12**<br><br>[123] | Patients with colorectal cancer A qualitative study of referral pathways and continuing care | Australia | Colorectal Cancer | Qualitative/Nineteen G.P.s/Four focus groups | Primary | The relationship between G.P. and the surgeon is essential in facilitating the referral pathway and the continuing role that can improve patients' situations. |

| | | | | | | |
|---|---|---|---|---|---|---|
| **Parkins;Edgar. (2011)/50** [124] | Comparison of the effectiveness of two enhanced glaucoma referral schemes | UK | Glaucoma | Quantitative/Statistical Analysis | Primary | to support referral decision-making by reducing costs while onward referral for refinement by accredited optometrists |

## Supplementary Table S3

Table S3:Papers studying the cause and effect and association analysis arranged in descending using citations per year

| **Author(s) /GC** | **Study the Association between** | **paradigm** | **Disease** | **Type of Referral** | **Findings** |
|---|---|---|---|---|---|
| (Sud et al. (2020)/152 [11] | Delay in The Referral pathway and the Mortality Rate | Modeling study | cancer | primary | For most solid cancers, delays to the treatment of 2–6 months will lead to substantial progress from curable to incurable cases. The overall impact of delays in referral is varied between cancers. |
| Lewis et al. (2018)/27 [18] | Delay in The Referral pathway and the Mortality Rate | Mixed | Musculo -skeletal & Cardiac | primary | low-level evidence suggests an association between early access to community outpatient services and the improvement of some patient outcomes. |
| Moller et al. (2015)/80 [69] | Delay in The Referral pathway and the Mortality Rate | Quantitative | cancer | primary | a clinically relevant association between the use of urgent referral for suspected cancer and increased risk of death among patients with cancer. |
| Santos et al. (2015)/23 [125] | Delay in The Referral pathway and the Mortality Rate | Quantitative | cancer -urology | cross/ surgery | The Referral Delay to the urologist increases the risk of mortality after surgery. Women patients had longer wait times and poorer survival. |
| Round et al. (2020)/16 [10] | Delay in The Referral pathway and the Mortality Rate | Quantitative | cancer | primary | Higher practice referrals for suspected cancer are associated with lower mortality for the four most common types of cancer. |
| Zhou et al. (2018)/37 [126] | Patient characteristics and Referral Patterns | Quantitative | cancer | primary | Fast-track referrals are less likely for cancers characterized by nonspecific presenting symptoms and patients with low cancer incidence demographic groups. |
| Hendijani; Bischak (2016)/5 [127] | Social relationships and Referral Patterns | Qualitative | general | primary | Self-confidence Physicians referred significantly fewer patients in a close relationship than in a centralized referral system. |
| Murchie et al. (2015)/13 [68] | Number of cases and case-mix and Referral Patterns | Quantitative | cancer | primary | Much apparent variation in GPs' use of suspected cancer referral pathways can be attributed to random case mix. |
| Mascia et al. (2015)/33 | Hospital referral networks on patient readmissions | Quantitative | general | primary | greater centrality is negatively associated with readmissions, whereas greater ego-network density increases the likelihood of readmission events. |

| | | | | | |
|---|---|---|---|---|---|
| [128] | | | | | |
| Jiwa et al. (2014)/8 [129] | Referral letters on scheduling hospital appointments | Quantitative | general | primary | The role of referral letter templates in improving specialist schedules. Not confirmed yet |
| Ringberg et al. (2013)/54 [58] | GPs' gender and specialist qualifications and referral Patterns | Quantitative | general | primary | Male GPs and specialists referred significantly less frequently to secondary care. And more for Radiologists. |
| Gordon et al. (2018)/2 [130] | Age of the Patient and Referral Patterns | Qualitative | Cancer | Primary | Referral patterns of newly diagnosed adolescent and young adult patients with cancer are associated Significantly with Patient age and PCP specialty. |

**Supplementary Table S4**

Table S4: Papers of interventions to solve referral problems using operational management models

| Authors | Article Title | Where | Disease | Model | Type of Referral | Conclusion |
|---|---|---|---|---|---|---|
| **An et al. (2018)/33** [118] | Analysis of the US patient referral network | USA | General | algorithms and methods from graph theory, network science, and statistics | General | To improve the referral network regarding healthcare system efficiency and physician workload, understanding network aspects of the most well-performing cases and investigating whether it is beneficial to expand or even impose such structures in other countries. |
| **Pires et al. (2013)/70** [131] | Assessing the Need for Referral in Automatic Diabetic Retinopathy Detection | General | Ophthalmology | Algorithm/Automatic Diabetic Retinopathy Detection | Primary | Algorithm to make that decision based on the fusion of results by meta-classification. The input of the meta classifier is the output of several lesion detectors, creating a powerful high-level feature representation for the retinal images. We explore alternatives for the bag-of-visual words (BoVW)-based lesion detectors, which critically depend on coding and pooling the low-level local descriptors. The final classification approach achieved an area under the curve of 93.4% using SOFT–MAX BoVW (soft-assignment coding/max pooling) without the need to normalize the high-level feature vector of scores |
| **Zhang et al. (2012)/75** [132] | Optimization of Prostate Biopsy Referral Decisions | General | Prostate cancer | Optimization of referral decision | Primary | estimate the optimal stopping prostate Biopsy time at 74 years of age |
| **Quellec et al. (2016)/38** [133] | Automatic detection of referral patients due to retinal pathologies through data mining | General | Ophthalmology | Automatic detection using data mining | Primary | In conclusion, a novel framework was presented for mining images and contextual information at adaptive granularity levels. This framework was successfully applied to eye pathology detection in a large screening dataset and could be generalized to other medical applications. |
| **Nijeweme-d'Hollosy et al. (2018)/25** [75] | Evaluation of three machine learning models for self-referral decision support on low back pain in primary care | General | Low back pain | Decision support using machine learning | Primary | The study showed promising results regarding the possibility of using machine learning in our CDSS design. The augmented-tree model performed better in classifying cases of lower back pain, but it still needs to be improved. Therefore, it is necessary to collect new cases, especially cases classified as self-care cases. |
| **Wang et al. (2020)/6** [134] | Hospital referral and capacity strategies in the two-tier healthcare systems | China | General | Stackelberg game with a queuing model | Reversed | A possible solution for how some patients can be effectively transferred from the CH to the PH, by considering a case in which the patients choose to either stay at the CH or shift downstream to the PH based on their expected utilities, we establish a three-stage Stackelberg game with a queuing model to capture the equilibrium strategy in terms of the CHP's referral rate and the PHP's capacity level in a two-tier Chinese healthcare system |

| | | | | | | |
|---|---|---|---|---|---|---|
| **Kobayashi et al. (2010)/55** [135] | A Geographical Information System Using the Google Map API for Guidance to Referral Hospitals | China | General | GIS Google Map | General | A GIS-based platform using OSS with the Google Map API. The system reduced the maintenance costs associated with other GIS-based platforms. Although this system has no underlying guarantees, we have experienced no problems guiding patients to outside clinics. |
| **Li et al. (2019)/5** [41] | Operational decision-making for a referral coordination alliance- When should patients be referred, and where should they be referred? | China | General | Simulation Experiments | ULH can transfer less urgent patients to the LLH (Reversed) | Patient referral problem in the two-stage treatment process in a referral alliance system containing one ULH and three LLHs. We investigate how to implement referrals from the view of operation to maximize the benefits of the entire healthcare system. We propose a threshold policy to make the Referral Decision - when patients should be referred, and five selection strategies to make Selection Decision – where patients should be referred. We built simulation models with five different selection strategies, and at the same time, we also developed a PSO-OCBA algorithm to find the best control threshold K for each selection strategy with the patients' average total waiting time of the four hospitals as the minimalizing objective |
| **Chen; Lin (2017)/11** [56] | Development of simulation optimization methods for solving patient referral problems in the hospital-collaboration environment | General | General | PSO to solve the problem. | Primary | By developing patient referral mechanisms, this study solved the problems related to the insufficiency of medical resources and the declining quality of medical services, resulting in a prolonged waiting time for MRI patients. This study analyzed two scenarios: without restrictions and with waiting time. The two contributions of this study are as follows.<br>(1)<br>The study constructed a simulated model for the case hospitals and verified and validated the proposed model<br>(2)<br>The study obtained a better number of MRI referrals in two simulated scenarios. By developing the three patient referral mechanisms with the PSO algorithm, this study determined a better number of referrals in Scenarios 1 (no constraints) and 2 (constraint: waiting time < 21 days), thereby minimizing the average waiting time in the three hospitals to within nine days and improving the quality of medical care. |
| **Li et al. (2021)/2** [136] | Pareto optimization for control agreement in patient referral coordination | China Shanghai | General | a multi-fidelity model-based simulation-optimization approach | Reversed | |

| | | | | | | |
|---|---|---|---|---|---|---|
| **Chen et al. (2016)/10** [55] | Patient referral mechanisms by using simulation optimization | General | General | Simulation Optimization | Primary | Two hospital model waiting time |
| **Stille et al. (2011)/17** [137] | Development and validation of a tool to improve paediatric referral/consultation communication | General | Pediatric | Iterative process Model | Primary | Communication elements are suitable for brief communication templates from pediatric PCPs to professionals. These a template may add value to communication between clinicians. |
| **Todd et al. (2018)/6** [138] | Text Mining and Automation for Processing of Patient Referrals | General | General | Data mining | Primary | This pilot study successfully demonstrated the potential for automating the assignment of referrals and provides a foundation for further work. This study also outlined a potential application of text mining and NLP to automate a hospital manual task to save human resources time. |
| **Zhou et al. (2021)/1** [42] | Coordinated appointment scheduling with multiple providers and patient-and-physician matching costs in specialty care | General | General | A stochastic optimization model | Primary | In a two-stage optimization problem, the algorithm efficiently solved the studied problem in a reasonable time. The optimal solution will assign patients to the most matched physicians if the matching cost dominates operational costs. Otherwise, the optimal solution would balance the workload of physicians as much as possible. |
| **Li et al. (2021)/1** [139] | A probabilistic linguistic evaluation-based multi-stage medical scheme selection process related to referral system | General | Lung cancer | Comparison analyses and simulation experiment | Primary | This paper provides a multi-stage medical scheme selection process to obtain a suitable scheme in the referral system. |
| **Li et al. (2019)/3** [140] | A Novel Multi-Attribute Group Decision-Making Method and Its Application in Solving the Downward Referral Problem in the Hierarchical Medical Treatment System in China | China | General | MAGDM/Mathematical Model | Reversed | The analytical results show that the new approach is applicable and operational in referral hospital selection, which provides convenience for a patient referred down in China's hierarchical medical treatment system. |
| **Maghsoud-Lou et al. (2017)/5** | Protocol-Driven Decision Support within e-Referral | General | Spinal problems | Semantic web Model | Primary | E-Referral process workflow as implemented by the SPER system |

| | | | | | | |
|---|---|---|---|---|---|---|
| [9] | Systems to Streamline Patient Consultation, Triaging, and Referrals from Primary Care to Specialist Clinics | | | | | |
| **Fischer et al. (2010)/12** [141] | Chicago: Using Evidence-Based Rules to Make Smarter Referrals | USA Chicago | General | Algorithms Prioritization | General | The Internet Referral Information System is a promising approach to increasing access to care, improving its quality, increasing operational efficiency and coordinating care among organizations. Its replication in other communities could significantly impact the quality and integration of the care delivered by healthcare safety-net providers. |
| **Zhong et al. (2019)/1** [142] | Bottleneck Analysis to Reduce Primary Care to Specialty Care Referral Delay | General | General | Mathematical Model | Primary | What emerges from the study is the need to integrate health information technology into medical personnel's workflow and redesign the process to allow effective communication, which will lead to improved care, efficiency, and satisfaction. |
| **Raptis et al. (2011)/3** [143] | Development of electronic web-based software for the management of colorectal cancer target referral patients | General | Colorectal Cancer | electronic web-based software | Primary | The use of web-based electronic software effectively facilitates and improves the quality of information communicated between users. The program assists clinicians in managing and organizing the daily tasks associated with patient care, including achieving goals opposed by the Ministry of Health. |
| **Spasic; Button. (2020)/0** [144] | Patient Triage by Topic Modeling of Referral Letters: Feasibility Study | General | Musculoskeletal | Using natural language processing and machine learning to automate the triage | Primary | The results established the feasibility of using natural language processing and machine learning to automate the triage of patients with knee or hip pain by analyzing information from their referral letters |
| **Adams et al. (2020)/0** [145] | Modeling improved efficiency in healthcare referral systems for the urban poor using geo-referenced health facility data: the case of Sylhet City Corporation, Bangladesh | Bangladesh | Maternal and emergency/critical care services | GIS-based modeling | General | This GIS-based modeling exercise is more convenient to the urban poor in terms of proximity and working hours |
| **Cusack et al. (2012)/0** | Breast clinic triage tool: Telephone | General | Breast Cancer | A tool was developed to triage referrals | Primary | Evaluation of a triage tool developed from local case mix analysis and similar publications has demonstrated a high |

| | | | | | | |
|---|---|---|---|---|---|---|
| [146] | assessment of new referrals | | | depending on the urgency | | level of accuracy for directing newly referred patients to the appropriate clinician and urgent assessment. It has been designed to be used friendly by administrative staff and has shown high acceptability to patients. |
| **Xiao et al. (2010)/0** [147] | Towards healthy China 2030: Modeling health care accessibility with patient Referral | China | general | two-step floating catchment area (2SFCA) model and its extensions | All | This paper proposes three conceptual models for patient referral and an innovative H2SFCA model to measure healthcare accessibility with the referral process between two levels of healthcare facilities. Applying it to regional and central hospitals in Beijing reveals that the H2SFCA model can identify areas in shortage of healthcare resources when patient referral is considered. More importantly, the case study has practical policy implications: implementing the patient referral will primarily benefit areas with sufficient health care resources, such as the urban core; exacerbate issues in areas devoid of health care resources (e. g., the suburbs); and likely polarize health inequality, primarily when health care facilities are unequally distributed. Therefore, caution is necessary to warrant the righteousness of the health policy reform in the Healthy China 2030 initiative by stressing flexibility in policy implementation. The increased scrutiny will eventually help to build a more efficient and equitable healthcare system. |
| **Francetic et al. (2021)/0** [119] | A network analysis of patient referrals in two district health systems in Tanzania | Tanzania | childhood illnesses and non-communicable diseases | Network analysis (or social network analysis) | All | showed the potential of using network analysis to assess patient referrals. The study results highlight the need for Tanzanian authorities to tackle the central issue of patient referrals from dispensaries. |

## Supplementary Table S5

Table S5: Papers of interventions to solve referral problems using management review arranged in descending using citations per year

| Authors | Article Title | where | Disease | Paradigm/Methodology/Sample Size | Type of Referral | conclusion |
|---|---|---|---|---|---|---|
| **Liddy et al. (2019)/46** [22] | A Systematic Review of Asynchronous, Provider-to-Provider, Electronic Consultation Services to Improve Access to Specialty Care Available Worldwide | North America, Brazil, Australia, Spain, and The Netherlands. | General | Qualitative/Systematic Review/Narrative 43 Article | Primary | E Consult systems are largely multiservice. While most new research examined outcomes related to patient and provider experience, cost measures require further development. |
| **Olayiwola et al. (2016)/90** [37] | Electronic Consultations to Improve the Primary Care-Specialty Care Interface for Cardiology in the Medically Underserved: A Cluster-Randomized Controlled Trial | General | Cardiology | Qualitative/Cluster-Randomized Controlled Trial/17 e-consultation &9 traditional | Primary | E-consultation referrals improved access to care, streamlined specialty referrals, and a potential solution for improving access to specialty care. |
| **Villeneuve et al. (2013)/126** [19] | A systematic literature review of strategies promoting early referral and reducing delays in the diagnosis and management of inflammatory arthritis | General | Inflammatory Arthritis | Mixed/Review/47 article | Primary | delay caring and potential solutions for patients with IA. A coordinated effort will be required by the rheumatology and primary care community to address these effectively |
| **Tuot et al. (2015)/72** [63] | Facilitators and barriers to implementing electronic referral and/or consultation systems: a qualitative study of 16 health organizations | General | General | Qualitative/Interviews/16 health organizations | Primary | systems (electronic referral and/or consultation) have great potential to streamline access to and enhance the coordination of specialty care delivery. |
| **Tobin-Schnittger et al. (2018)/20** [23] | Improving quality of referral letters from primary to secondary care: a literature review and discussion paper | General | General | Qualitative/Narrative Review/18 Article | Primary | Electronic referrals, peer feedback, templates, and mixed interventions are four types of interventions. Intervention Mix provides tangible improvements in content and appropriateness |
| **Mansell et al. (2011)49** [20] | Interventions to reduce primary care delay in cancer referral: a systematic review | General | Cancer | Mixed/systematic Review/22 papers | Primary | using a Single Intervention has no evidence that directly reduces primary care delay. |
| **Blank et al. (2014)/35** [13] | Referral interventions from primary to specialist care: a systematic review of international evidence | General | General | Mixed/Review/140 article | Primary | the whole-systems approach is needed because the introduction of interventions in primary care is often just the starting point of the referral process. In addition, |

| | | | | | | |
|---|---|---|---|---|---|---|
| **Rea et al. (2018)/16** [148] | Shared Care: Using an Electronic Consult Form to Facilitate Primary Care Provider-Specialty Care Coordination | General | Children | Qualitative/Survey | Primary | an electronic referral and consultation system was feasible and provided timely access to specialty care but did not impact referral volume. |
| **Dusheiko; Gravelle. (2018)/14** [149] | Choosing and booking attending? Impact of an electronic booking system on outpatient referrals and non-attendances | General | General | Quantitative/Statistical Analysis/7700 practices | Primary | practices that increased their use of the Choose and Book C&B system experienced a reduction in the proportion of referred patients failing to attend. They also reduced their referral rates |
| **Greenwood-Lee et al. (2018)/13** [14] | A categorization of problems and solutions to improve patient referrals from primary to specialty care | General | General | Qualitative/Review/106 articles | Primary | interventions may produce improvements. Overlap and interconnectedness between interventions create potential synergies and conflicts among co-implemented interventions. |
| **Long et al. (2018)/13** [150] | Using behavior change and implementation science to address low referral rates in oncology | Australia | Cancer | Qualitative/Focus groups and interviews/19 healthcare professionals | Cross/ Genetics | While our approach did not improve referrals, we have deepened our understanding of barriers to referral and approaches to low-frequency conditions. |
| **Borooah et al. (2013)/26** [151] | Using electronic referral with digital imaging between primary and secondary ophthalmic services: a long-term perspective analysis of regional service redesign | General | Ophthalmology | Quantitative/Long term prospective analysis/ | Primary | Using IT infrastructure improves communication between primary and secondary care. That promotes the effective use of limited outpatient capacity by retaining non-progressive, asymptomatic pathology patients in the community while fast-tracking patients with the sight-threatening disease |
| **Othman; Menon. (2019)/8** [152] | Developing a nationwide spine care referral programme on the WhatsApp messenger platform: The Oman experiment | Oman | Spine | Model/WhatsApp Group/ 8 regional hospitals in Oman | Primary | Spine care service in Oman has benefitted immensely from mobile applications for patient Referral to the tertiary care facility. The response time has reduced considerably, and cases and emergency care prioritization have also improved dramatically. The outpatient waiting times have also shown a significant reduction due to the use of this technology. |
| **La Rocca; Hoholm. (2017)/13** [7} | Coordination between primary and secondary care: the role of electronic messages and economic incentives | General | General | Qualitative study/ Interviews/17 employees | General | that work jointly. Incentives and instruments are likely to favor the collaboration between primary and secondary care. |

| | | | | | | |
|---|---|---|---|---|---|---|
| **Vos et al. (2020)/4** [153] | The influence of electronic health record use on collaboration among medical specialties | Germany | General | Qualitative/Case study at five outpatient clinics | General | EHRs' collaboration in hospitals. That requires organizational, technical, and behavioral adaptations. That enhances the EHR and its use for effective collaboration. |
| **Vimalananda et al. (2019)/6** [154] | Tools to Improve Referrals from Primary Care to Specialty Care | General | General | Mixed/Adjusted odds ratios/ 2533 clinicians from 13 medical specialties | Primary | referral templates may help improve the collaboration with primary care and be broadly applied to improve referrals |
| **Common et al. (2018)/8** [155] | Reducing Wait Time for Lung Cancer Diagnosis and Treatment: Impact of a Multidisciplinary, Centralized Referral Program | General | Lung Cancer | Qualitative/Retrospective cohort study/Statistical analysis/133 patients | Primary | A collaborative, centralized referral program helps to reduce wait time for diagnosis and treatment of lung cancer. |
| **Wahlberg et al. (2013)/17** [156] | Practical health cooperation - the impact of a referral template on quality of care and health care cooperation: study protocol for a cluster randomized controlled trial | General | dyspepsia, chest pain, suspected colonic malignancy, and chronic obstructive pulmonary disease | Qualitative/Statistical Analysis/Cluster Randomized Trial/14 community GP | Primary | Using a referral template in this communication will lead to a measurable increase in healthcare quality delivered. |
| **Kinahan et al. (2017)/6** [157] | Promoting the Shared-Care Model for Adolescent and Young Adults with Cancer: Optimizing Referrals and Care Coordination with Primary Care Providers | General | Cancer | Qualitative/Education video Module and an online survey | Primary | Best practice advisory (BPA) or "stopgap" intervention: Providers that completed the intervention survey agreed/strongly agreed that the shared-care model is desirable |
| **Delva et al. (2012)/17** [28] | Referral of elderly cancer patients to specialists: Action proposals for general practitioners | General | Cancer | Mixed/Review/Thirty eligible articles | Primary | Focused action proposals are presented to improve learning and optimize the referral process. |
| **Nabelsi et al. (2019)/5** [5] | Improving the Referral Process, Timeliness, Effectiveness, and Equity of Access to Specialist Medical Services Through Electronic Consultation: Pilot Study | General | General | Model/e Consult service | Primary | The E- Consult platform proves effective, efficient, and well-received by both patients and physicians. Reduce wait times significantly. |
| **Wahlberg et al. (2017)/8** [158] | The effect of referral templates on outpatient quality of care in a hospital setting: a cluster randomized controlled trial | General | General | Mixed/A cluster randomized controlled trial/14 community GP surgeries | Primary | after implementing referral templates at the general practitioner/hospital interface. Limited intervention uptake at GP surgeries and inconsistencies in outcome assessment hindered the results. |

| | | | | | | |
|---|---|---|---|---|---|---|
| **Eskeland et al. (2017)/4** [159] | Assessment of the effect of an Interactive Dynamic Referral Interface (IDRI) on the quality of referral letters from general practitioners to gastroenterologists: a randomized cross-over vignette trial | Norway | Gastroenterologist | Quantitative/Long term prospective analysis/statistical/25 GPs | Primary | Diagnose-specific checklists to improve the quality of referral letters significantly and reduce the variance of the TPS |
| **Mariotti et al. (2013)/7** [160] | Improving referral activity on primary-secondary care interface using an electronic decision support system | General | General | Quantitative/1126 records 62 PCPs | Primary | feedback and ERSS used for improving referral activity and agreement between PCPs and specialists. |
| **Wright et al. (2015)/4** [161] | An evidence-based referral management system: insights from a pilot study | General | General | Model/A pilot study /A mixed methods | Primary | the new referral management system. Were preliminary effectiveness |

# Supplementary Table S6

Table S6: Articles focusing on the quality management of the referral pathway ranked in descending order by the article citation number per year

| **Authors** | **Article Title** | **where** | **Disease** | **Paradigm/Methodology/Sample Size** | **Type of Referral** | **conclusion** |
|---|---|---|---|---|---|---|
| **Baughan et al. (2011)/69** [67] | Urgent suspected cancer referrals from general practice: audit of compliance with guidelines and referral outcomes | Scotland | Cancer | Quantitative/Prospective audit | Primary | There is a wide variation in GP referral rates for suspected cancer, primarily referrals for younger people. Suggesting factors outside referral guidelines alert GPs to the possibility of cancer |
| **Nicholson et al. (2016)/27** [162] | International variation in adherence to referral guidelines for suspected cancer: a secondary analysis of survey data | Six Countries | Cancer | Qualitative/Secondary Analysis of Survey Data/five clinical vignettes | Primary | Cancer guideline varies between developed nations. Poor guideline adherence may reduce outstanding diagnostic efforts. |
| **Kim et al. (2015)/31** [163] | Care coordination between specialty care and primary care: a focus group study of provider perspectives on strong practices and improvement opportunities | General | General | Qualitative/Focus Group Design/48 sessions | Primary | Improve care coordination, using mutual understanding and respect developed between SCPs and PCPs through relationship-building efforts. |

| | | | | | | |
|---|---|---|---|---|---|---|
| **Roy; Anjum. (2018)/13** [164] | The two-week wait - a qualitative analysis of suspected head and neck cancer referrals | General | Head and Neck Cancer | Qualitative /Retrospective Examination/141 Urgent Referrals | Primary | The predictive value of the two-week wait referral of the head and neck cancer was low, suggesting significant inappropriate pathway use. |
| **Sandager et al. (2019)/9** [165] | Cancer patients' experiences with urgent referrals to cancer patient pathways | General | Cancer | Qualitative/cross-sectional questionnaire surveys/2,256 individuals | Primary | CPP-referred cancer patients have better experiences of pre-diagnosis cancer care than non-CPP-referred patients. they were attributed to shorter diagnostic intervals and the absence of quality deviations among CPP patients. |
| **Kostopoulou et al. (2019)/9** [166] | Referral Decision-Making of General Practitioners: A Signal Detection Study | England | Lung Cancer | Quantitative/Signal Detection Study/44 vignettes | Primary | They are improving the evidence base for cancer referral decisions by Increasing discrimination. |
| **Guassora et al. (2015)/19** [167] | Preparing general practitioners to receive cancer patients following treatment in secondary care: a qualitative study | General | Cancer | Qualitative/Focus Groups | Reversed | Improve healthcare professionals' engagement and coordination of care across organizational boundaries. |
| **Jack et al. (2012)/26** [6} | Something's missing here: a look at the quality of rheumatology referral letters | General | Rheumatology | Qualitative/Focus Groups/135 referral letters | Primary | A standardized referral template was developed to overcome the lack of information in the Referral letter. |
| **Valentin-Lopez et al. (2012)/25** [66] | Assessment of a rapid referral pathway for suspected colorectal cancer in Madrid | Madrid | Colorectal Cancer | Quantitative/Statistical analysis/272 patients | Primary | The rapid referral pathway reduced the waiting time for colonoscopy and overall waiting time for definitive treatment. It appeared to be an effective strategy for diagnosing CRC in its early stages. |
| **Harahsheh et al. (2021)/2** [168] | Promoting Judicious Primary Care Referral of Patients with Chest Pain to Cardiology: A Quality Improvement Initiative | USA | Cardiology | Qualitative/Quality Improvement Model/421 patient | Primary | Feasible, effective, and safe reduce low-probability cardiology referrals for children presenting to primary care practices with chest pain. |
| **Esteve-Matali et al. (2021)/2** [169] | Understanding How to Improve the Use of Clinical Coordination Mechanisms between Primary and Secondary Care Doctors: Clues from Catalonia | Catalonia | General | Qualitative/Cross-Sectional Online Survey/3307 PC and SC doctors | Primary | to identify the difficulties and factors affecting the use of several cross-level clinical coordination mechanisms |
| **Zhang et al. (2018)/6** [170] | Key nodes affecting patient satisfaction in a cross-regional referral service process: an empirical analysis study in Sichuan | China | General | Mixed/Questionnaire Survey/Correlation analysis and logistic regression/110 patients | All | key nodes affecting patient satisfaction were "transferring service at the primary-level hospital" and "admission service at the higher-level hospital." Furthermore, the efficacy of the referral services is determined |

| | | | | | | |
|---|---|---|---|---|---|---|
| | | | | | | by the gatekeepers' management of the referral system at the primary-level hospital and the allocation and management of bed resources at the higher-level hospital. |
| **Rathnayake; Clarke. (2021)/1** [17] | The effectiveness of different patient referral systems to shorten waiting times for elective surgeries: systematic review | General | Surgical care | Qualitative/Narrative Review/6 Articles & 3 Reviews | Primary | Interventions could be implemented in the referral process for an adult elective surgery to improve waiting times, specifically for high-income countries. |
| **Murray et al. (2020)/2** [171] | Delphi method to identify expert opinion to support children's cancer referral guidelines | General | Children Cancer | Qualitative /Delphi method/25 draft statements for review/88 pediatric hematologist &oncologist | Primary | This expert opinion should help to optimize suspected cancer referral in children, subsequent outcomes, and experiences for patients and families |
| **Van den Bogaart et al. (2020)/2** [172] | Referral decisions and their predictors related to orthopedic care. A retrospective study in a novel primary care setting | General | Orthopedic Care | Quantitative/Retrospective Study/logistic regression modeling | Primary | PC+ aims to prevent unnecessary referrals to hospital care. |
| **Cain; Collins. (2018)/4** [173] | Using quality improvement to improve internal and external coordination and referrals | General | General | Project/Quality Improvement Model | General | Identifying organizational gaps, priorities, and improvement projects using Quality Improvement Plan |
| **Ilboudo et al. (2012)/7** [174] | Assessment of providers' referral decisions in Rural Burkina Faso: a retrospective analysis of medical records | Burkina Faso | Malaria and Pneumonia | Mixed/A record review of twelve months of consultations | Primary | Low adherence to the National Guidelines founded. Health center providers need to be better trained in the diagnostic process and disease management in rural Burkina Faso. |
| **Kreiter et al. (2012)/7** [175] | Referral Rate to Oncologists and its Variation by Hospital for Colorectal Cancer Patients | General | Colorectal Cancer | Mixed/Focus Group | Primary | The group Discusses interventions to improve oncologist-consult rates, particularly for stage II rectal cancer patients. |
| **Alberti et al. (2019)/2** [176] | Optimizing patient referral and center capacity in the management of chronic hepatitis C: Lessons from the Italian experience | Italia | Hepatitis | Qualitative/Delphi study | Primary | This Delphi study considers the need for an increased role of GPs, increasing center capacity, particularly medical personnel in outpatient centers, and more effective liaison between Hub centers and healthcare professionals to eradicate HCV. |
| **Arain et al. (2020)/1** [177] | Specialist LINK and primary care network clinical pathways - a new approach to patient Referral: a cross-sectional survey of awareness, utilization, and usability among family physicians in Calgary | Calgary | General | Qualitative/E Cross-Sectional Survey | Primary | Most of the respondents in Calgary and the area were aware of Specialist LINK, and a large proportion of them used it to access advice for their patients. |

| | | | | | | |
|---|---|---|---|---|---|---|
| **Ho et al. (2016)/2** [178] | Optimizing the pre-referral workup for gastroenterology and hepatology specialty care: consensus using the Delphi method | General | Gastroenterology and Hepatology | Qualitative/Delphi method | Primary | Considering externally developed referral guidelines using a consensus-building process leads to significant local tailoring and adaption. |
| **Rodrigues et al. (2021)/0** [179] | Evaluation of the quality of referral letters: experience of a Brazilian oral medicine service | Brazil | Oral Medicine Service | Mixed/Quality Improvement Model/500 Referral letters | Primary | Only 58 Referral letters were classified as good and three as excellent, Among the 500 referral letters analyzed in this study. |
| **Fernandez-Mendez et al. (2020)/0** [180] | Improvement of the Efficiency and Completeness of Neuro-Oncology Patient Referrals to a Tertiary Center Through the Implementation of an Electronic Referral System: Retrospective Cohort Study | UK | Neuro-Cancer | Quantitative/Retrospective Cohort Study/248 patients | Primary | Decrease waiting times and improve the completeness of information using an electronic, Web-based for neuro-oncology referrals. |
| **Kirby et al. (2018)/13** **[181]** | Using Clinical Decision Support to Improve Referral Rates in Severe Symptomatic Aortic Stenosis: A Quality Improvement Initiative | General | Cardiology | Project/Quality Improvement Model | Primary | Evaluation of the use of a clinical decision support system. to improve specialist referral rates for patients with severe aortic stenosis. |
